# Genetic architectures of brain-related traits are shaped by strong selective constraints

**DOI:** 10.64898/2026.03.22.713538

**Published:** 2026-03-25

**Authors:** Huisheng Zhu, Yuval B. Simons, Jeffrey P. Spence, Guy Sella, Jonathan K. Pritchard

**Affiliations:** 1 Department of Biology, Stanford University, Stanford, CA,; 2 Department of Genetics, Stanford University, Stanford, CA,; 3 Section of Genetic Medicine, University of Chicago, Chicago, IL,; 4 Department of Human Genetics, University of Chicago, Chicago, IL,; 5 Institute for Human Genetics, University of California, San Francisco, CA,; 6 Department of Epidemiology & Biostatistics, University of California, San Francisco, CA,; 7 Department of Biological Sciences, Columbia University, New York City, NY,; 8 Program for Mathematical Genomics, Columbia University, New York City, NY

## Abstract

Genome-wide association studies (GWAS) have identified hundreds of significant loci for psychiatric disorders, yet the strength of these associations remains modest compared to other human complex traits with similar numbers of hits. Whether this pattern reflects statistical artifacts or real biological differences — and, if the latter, what underlies it — remains unclear. In addition to psychiatric disorders, we find that other traits with functional enrichment in the central nervous system (CNS), whether binary or quantitative, also share similar genetic architectures, characterized by GWAS hits of limited statistical significance and generally higher allele frequencies. To robustly compare traits that differ in GWAS statistical power, we demonstrate how binarizing a quantitative trait reduces power. This loss of power can be replicated by a matched “effective sample size” on the liability scale. After matching “effective sample sizes”, we show that CNS-enriched traits have large mutational target sizes, with contributing variants and genes experiencing stronger selection than those for other traits. Our findings reveal heterogeneity among diseases and provide insights into traits that more effectively capture fitness-relevant processes. More broadly, our results suggest that the genetic architectures of complex traits are shaped by the tissues through which these traits are mediated.

## 1 Introduction

Many, if not most, traits in humans and in other species are genetically complex, with heritable variation spread over myriad variants throughout the genome. Complex traits have been studied for more than a century [1, 2], but only recently has it become possible to systematically dissect the genetic basis of their heritable variation. Over the past two decades, genome-wide association studies (GWAS) in humans have identified tens of thousands of robust associations between genetic variants and a wide array of quantitative traits and complex diseases [3, 4].

One intriguing observation from GWAS is that the genetic architectures of complex traits can vary widely. (Here, we use the term “architecture” to refer to the number of causal variants and their joint distribution of allele frequencies and effect sizes [4]. Often, we focus on the subset of approximately independent variants that exceed genome-wide significance in GWAS.) Notable trait variation in genetic architectures includes differences in the number and magnitude of significant associations detected within the same cohort [5, 6], as well as differences in the distributions of effect sizes [7] and allele frequencies [8] among significant variants.

Understanding the causes and determinants of this variation in architecture is of both practical and foundational importance. From an applied perspective, a trait’s genetic architecture determines the performance of studies aimed at mapping its genetic basis, as well as the accuracy of phenotypic predictions derived from these studies [9–12]. From a foundational perspective, the architecture of a trait reflects the evolutionary forces that shape heritable variation in natural populations and largely determines the genetic and phenotypic adaptive response to changes in selection pressures [13].

Recent work from Simons *et al*. tackled this problem from an evolutionary perspective [14, 15]. Motivated by extensive evidence that many quantitative traits are subject to stabilizing selection, with fitness declining with displacement from an optimal trait value [13, 16, 17], and that genetic variation affecting one trait typically affects many others [9, 18, 19], they modeled how selection on variants arises from stabilizing selection in a multi-dimensional trait space [14]. Their model also incorporated the effects of mutation and genetic drift. Given a demographic history and mutation rate per site, the genetic architecture of a trait in this model depends on two parameters and a distribution: the mutational target size (i.e., the number of sites in which a mutation affects the trait), the heritability per site in the target, and the distribution of selection coefficients for newly arising mutations in the target. Fitting this model to the 95 quantitative traits with more than a hundred approximately independent hits in the UK Biobank, they find that this model of pleiotropic stabilizing selection provides a better fit to the architectures of these 95 traits than previous ones [20–25] and that the distributions of selection coefficients are fairly similar across traits. Consequently, most of the variation in GWAS results across traits is driven by differences in the heritability per site in the target [15]. However, it remains unclear whether this result holds true for complex traits in general.

Several studies have suggested that the genetic architectures of psychiatric disorders and more generally of brain-related traits share characteristics that set them apart from other complex traits. We call a trait “brain-related” if variation in its value is largely mediated by genetic effects acting in cells of the central nervous system (CNS), and we identify such traits with stratified LD score regression (S-LDSC), testing for enrichment of SNP heritability in open chromatin regions of CNS cells [26, 27]. Brain-related traits are consistently found to be among the most highly polygenic traits examined in GWAS based on a variety of polygenicity measures [25, 28–30], suggesting that they have large mutational target sizes. Additionally, work by Holland *et al*. showed that under a point-normal model, non-null variant effect sizes for brain-related traits have variances that are orders of magnitude smaller than those for other complex traits [31]. When modeling the architecture with a flexible number of normal components, the effect sizes for brain-related traits were well approximated by a single normal distribution, whereas those for other complex traits typically required a mixture of two or more normals with different scales [32]. These lines of evidence suggest that brain-related traits arise from a large number of causal variants with relatively evenly distributed contributions to phenotypic variance.

Another consideration is that the traits Simons *et al*. examined were all quantitative; should we even expect complex diseases, including psychiatric disorders, to share similar properties in their genetic architectures? Intuition might suggest that genetic variation affecting complex diseases would be under directional selection, namely, that alleles that decrease the risk of disease would be selected for and alleles that increase this risk would be selected against. In contrast, the pleiotropic stabilizing selection model postulates that when mutations arise, they are always selected against, and for any given selection coefficient, they are equally likely to increase or decrease liability. Several recent studies support the latter view, indicating that, at least for common genetic variation, the architecture of complex diseases is predominantly shaped by pleiotropic stabilizing selection [7, 33, 34]. These findings therefore justify extending the Simons model to complex diseases.

Thus, we wanted to test whether the Simons model fits brain-related traits, including psychiatric disorders, and to explore how, and whether, these traits differ from other categories of traits. Do brain-related traits have unique genetic architectures? If so, what are the evolutionary causes that set brain-related traits apart? Here we set out to answer these questions.

We analyzed GWAS results across a wide range of complex traits and discovered that brain-related traits have unique features, with significant hits narrowly exceeding the significance threshold and enriched at higher allele frequencies. After adjusting for statistical power, which is influenced by sample size differences and the binary nature of diseases, we conclude that psychiatric disorders, along with other brain-related quantitative traits, have large mutational target sizes, and their underlying variants and genes are experiencing stronger selection.

## 2 Results

**Figure 1:**
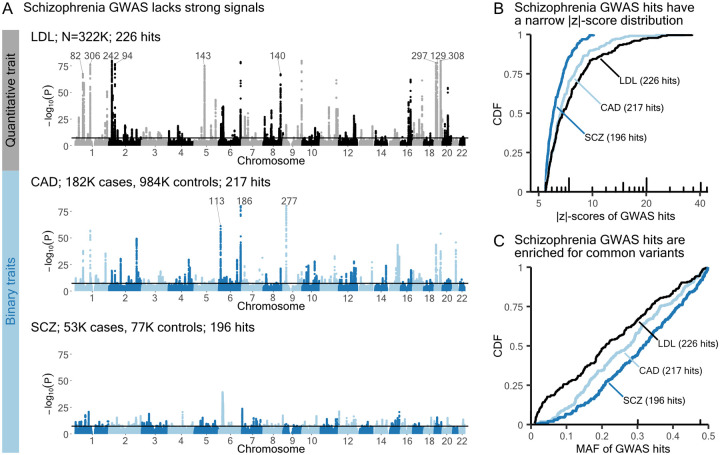
Schizophrenia GWAS hits narrowly exceed the significance threshold. **A)** Manhattan plots for three example traits. The y-axes of all three plots are restricted to the range [0, 80]. Significant associations beyond this range are labeled with the most significant p-value. Independent hits are obtained from GCTA-COJO [38], and hits with MAF below 1%, in the HLA region, or of low imputation quality are excluded (Methods). **B)** Cumulative distributions of COJO hit absolute z-scores. **C)** Cumulative distributions of COJO hit MAF. CAD: coronary artery disease; SCZ: schizophrenia.

Substantial variation in genetic architecture revealed by GWAS can be exemplified with three contrasting traits: a non-brain-related quantitative trait — low-density lipoprotein cholesterol (LDL) [35]; a non-brain-related complex disease — coronary artery disease [36]; and a brain-related complex disease — schizophrenia [37]. While these GWAS yield similar numbers of approximately independent hits (Methods), the significant associations for schizophrenia surpass the genome-wide significance threshold by only a small margin (Figure 1A).

Differences in strength of significant associations are more readily apparent in the cumulative distributions (CDFs) of |z|-scores of their approximately independent hits (Figure 1B). Schizophrenia, which lacks strong signals, has a steeper CDF curve. When we instead plot the cumulative distributions of MAF, a new piece of information emerges: schizophrenia GWAS hits are generally higher frequency (Figure 1C).

Plots in the style of Figures 1B and C provide useful summaries of genetic architectures across traits. Based on these results, we wanted to understand why schizophrenia has such a distinct genetic architecture.

### The distinct genetic architectures of brain-related traits

We asked whether these differences in architecture are seen more generally between brain-related and other complex traits. To this end, we considered the 151 quantitative traits in the UK Biobank with at least 20 approximately independent hits and SNP heritability above 5%, as well as 13 human complex diseases with publicly available case-control GWAS summary statistics that meet these same criteria (Supplementary Tables 1–2, Methods).

**Figure 2:**
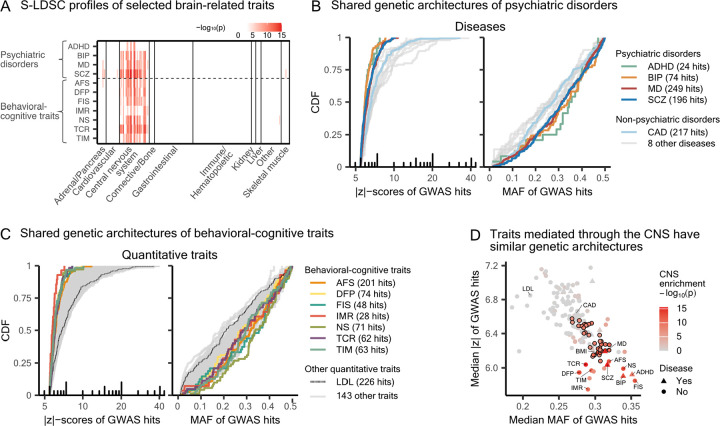
Traits with functional enrichment in the CNS share similar GWAS hit patterns. **A)** Functional enrichment analyses with S-LDSC [26, 27] on selected traits. Each row consists of 220 cell types from 10 categories, colored by $-{log}_{10}(p)$ for the coefficient τ if significant after Bonferroni correction. The gradient scale is bounded above at 15. Results on all other traits are in Figures S4 to S6. **B)** Contrasting the GWAS hits for psychiatric disorders with those of nine other complex diseases. The full list of diseases can be found in Supplementary Table 2. **C)** Contrasting the GWAS hits for behavioral-cognitive traits with those of 144 other quantitative traits. The full list of quantitative traits is in Supplementary Table 1. **D)** GWAS hits for traits enriched for the CNS have higher median MAF and lower median |z|-score. Each trait included in this study is colored by the combined p-value for CNS enrichment, computed following the aggregate Cauchy association test (ACAT) [41] (Methods). The points corresponding to the 36 body composition–related traits are outlined with black borders. ADHD: attention deficit hyperactivity disorder; BIP: bipolar disorder; MD: major depression. AFS: age first had sexual intercourse; DFP: duration to first press of snap-button in each round; FIS: fluid intelligence score; IMR: number of incorrect matches in round; NS: neuroticism score; TCR: time to complete round; TIM: mean time to correctly identify matches. BMI: body mass index.

We classified these traits as brain-related or non–brain-related by applying a significance threshold of 0.05/(10 × 164) to the meta-analyzed S-LDSC [26, 27] p-values for CNS enrichment (Methods). This classification yielded 50 brain-related quantitative traits, including seven behavioral-cognitive traits, and 101 non-brain-related quantitative traits (Figures S4 and S5). We also identified four brain-related diseases — all of which are psychiatric disorders — and nine non–brain-related diseases (Figure S6). Among brain-related traits, psychiatric disorders and behavioral-cognitive traits show the strongest CNS-specific enrichment (Figure 2A).

As in the schizophrenia example, we find that brain-related traits, including both quantitative traits and diseases, generally have GWAS hits spanning a narrower range of statistical significance and are enriched for higher MAF compared with other complex traits (Figures 2B and C). By contrast, neurological diseases — including Alzheimer’s disease, Parkinson’s disease, and multiple sclerosis — are classified as non–brain-related, and do not exhibit the genetic architecture observed for brain-related traits (Figure S7).

At a fixed sample size, the expected strength of association for a given variant i in GWAS is determined by its MAF $p_{i}$ and squared effect size $\beta_{i}^{2}$; more precisely, $z_{i}\propto 2p_{i}\left(1-p_{i}\right)\beta_{i}^{2}$ (see, e.g., supplementary information of [39]). Despite being enriched for common variants, GWAS hits for brain-related traits show narrower distributions of |z|-scores. This apparent contradiction is attributable to their narrower distributions of squared effect sizes relative to other complex traits (Figure S8), echoing previous findings [31, 32].

The narrow distributions of |z|-scores, elevated MAF, and small effect sizes place the genetic architectures of brain-related traits at an extreme among complex traits (Figures 2D, S9 and S10). Traits mediated through other tissues, including the adrenal gland and pancreas, kidney, and skeletal muscle, also cluster in this parametrization of architecture, but none are set apart from other traits as much as those mediated by the CNS. If a trait is mediated by the CNS as well as cells in other tissues, we might expect its genetic architecture to be intermediate between traits exclusively enriched for the CNS and non-brain-related traits. This is what we see for body composition-related traits (Figures 2D and S11B), which are partially mediated by the CNS through appetite control and preference for food intake [40], but are also mediated by other tissues (Figure S11A). Here, we focus on the pronounced differences between brain-related traits and other traits, but we revisit the potential roles of other tissues on trait genetic architectures in the Discussion.

Importantly, we wanted to ensure that these patterns were not driven by uncontrolled confounding in population-based GWAS of these traits. By comparing results from population-based and family-based association studies, previous studies have suggested that confounders including population stratification, indirect genetic effects, and assortative mating, can aggregate across the genome to bias heritability and genetic correlation estimates [42–45]. However, our analyses focus on GWAS hits, and we expected confounding effects at these strong-signal sites to be minor [45, 46]. To check this, we used a GWAS of birth coordinate as a negative control where most signals, except a few protective against allergies [47], are expected to be driven by confounding. MAF of GWAS hits for birth coordinate traits have a cumulative distribution that is distinct from any of the traits we considered (Figure S12).

While birth coordinates serve as a negative control trait, we were intrigued by urinary biomarkers, some of which were classified by our pipeline as brain-related and are less obviously prone to confounding from population stratification, assortative mating, or indirect genetic effects as compared to behavioral-cognitive traits. We noticed that creatinine and sodium levels in urine are classified as brain-related, while the level of creatinine in blood is not (Supplementary Table 1, Figure S13A). This is because in clinical practice, serum creatinine assesses the kidney’s normal function of eliminating waste products from muscle metabolism, whereas urinary creatinine is often used to normalize other urinary biomarkers, given that creatinine excretion is kept at a relatively constant rate [48]. Thus, unadjusted urinary creatinine and sodium concentrations are indicative of hydration status and urine flow rate, which involves coordination between the brain and bladder [49]. GWAS results of these urinary traits more closely resemble those of other brain-related traits than those of serum creatinine, which again supports that traits mediated through the CNS share similar genetic architectures (Figure S13B). Overall, this suggests that confounding is not driving the distinct patterns we see for the brain-related traits in our analyses, including the behavioral-cognitive ones.

### Brain-related traits retain distinct architectures after adjusting for statistical power

To ensure a rigorous comparison between brain-related and non-brain-related traits, we wanted to account for the binary nature of disease traits and for variation in sample size across traits. Both factors influence GWAS statistical power and, consequently, the learned genetic architectures.

**Figure 3:**
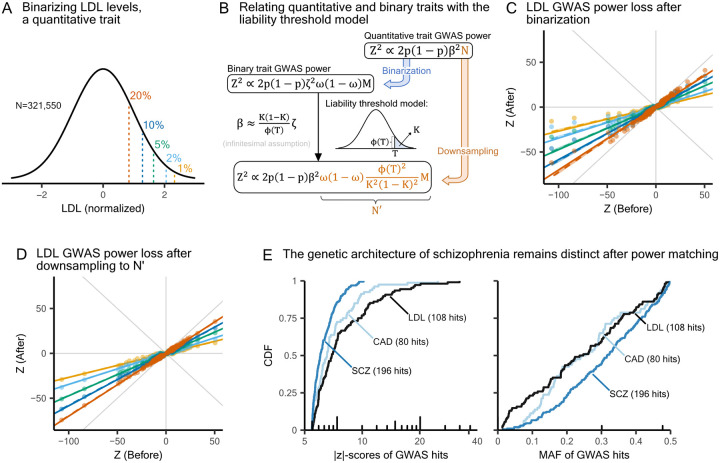
Calibrating GWAS statistical power between quantitative traits and binary traits. **A)** Binarizing LDL with varying prevalence in the upper tail. LDL levels were measured in unrelated White British individuals from the UK Biobank. The original phenotypic distribution was standardized using rank-based inverse normal transformation. **B)** Derivation of the sample size needed on the liability scale to reach equivalent power of a case-control GWAS. MAF is denoted by p. We transferred effect sizes from log odds ratio scale (ζ) to continuous scale (β) using the liability threshold model. For details, see Supplementary Notes. **C)** Deflation of LDL GWAS hit z-scores after binarizing. Different colors reflect the threshold used to binarize traits, as shown in panel A. Solid lines represent predicted slopes, and dashed lines indicate fitted slopes. Gray lines in the background are y=0,x=0 and y=±x. **D)** Deflation of LDL GWAS hit z-scores after downsampling to matched sample sizes. Color choices, line styles, and background lines are identical to those in panel C. **E)** Reducing coronary artery disease and LDL GWAS power to match with the current effective sample size of schizophrenia GWAS, $N^{\prime }=192,273$ (Methods).

We begin by examining binary traits, for which GWAS is typically conducted using logistic regression rather than the linear regression applied to quantitative traits. To study the impact of this difference, we converted quantitative traits into binary traits by applying a cutoff to the phenotypic values, a procedure we refer to as “binarizing” a trait. Our goal was to determine sample sizes that yield equivalent GWAS power between the binarized and the original continuous versions of essentially the same trait. Specifically, we binarized LDL levels using thresholds corresponding to varying population prevalences of the resulting binary trait (Figures 3A and S14A, Methods).

Based on the classical liability threshold model [50], an individual’s liability is a quantitative trait that relates to their genotype through the standard additive model, and individuals with liability exceeding a threshold T develop the disease. This model allows us to map case-control GWAS results for complex diseases to the corresponding GWAS results on the underlying liability, which is typically unobserved. Assuming variant effect sizes are sufficiently small, the power to identify a variant in a case-control study with a sample size of M individuals, with a proportion ω of cases and (1-ω) of controls, is approximately equal to the power to identify that variant in a GWAS of liability with effective sample size

(1) $$N^{\prime }\approx M\omega \left(1-\omega \right)\frac{\phi (T{)}^{2}}{K^{2}(1-K{)}^{2}},$$

where K is the population prevalence of the disease, and ϕ(⋅) denotes the standard normal density (Figure 3B, Supplementary Note).

In our analysis, we can treat the original trait as the underlying liability for the binarized trait. Accordingly, we used independent hits from the original quantitative LDL GWAS and evaluated the extent to which their z-scores were deflated in case-control GWAS across different binarization thresholds (Figures 3C and S14B). We also performed quantitative LDL GWAS on a subsample whose size was determined by Equation 1 to match the reduction in power caused by binarizing for each threshold we used (Figure 3D). Both binarizing and downsampling are expected to approximately linearly deflate z-scores with

(2) $$Z_{\text{after}}\approx \sqrt{\frac{N^{\prime }}{N}}Z_{\text{before}},$$

where N is the sample size of the original quantitative trait GWAS (Supplementary Note).

Figures 3C and D share the same set of predicted lines as given by Equation 2, and our theory accurately predicts the effects of both binarizing and downsampling. Variants of large effect deviate from the linear relationships in Figure 3C and S14B but not in Figure 3D, because Equation 1 relies on the infinitesimal assumption, whereas Equation 2 does not (Supplementary Note).

We repeated this analysis with five other medically relevant quantitative traits in the UK Biobank to ensure that these results were not unique to LDL (Figures S15 to S19). Unexpectedly, many traits exhibit asymmetrical power reduction when binarizing in the lower tail compared to the upper tail. For example, BMI shows more deflation than expected when binarizing in the lower tail, and less deflation in the upper tail (Figure S15). This pattern suggests that the same set of variants have, on average, stronger effects in high-BMI individuals than in low-BMI individuals. One possible explanation for this tail asymmetry is genetic epistasis, in which the magnitude of a variant’s effect varies depending on an individual’s genetic background, resulting in larger effects at one end of the liability distribution compared to the other. Gene-environment interaction is another plausible mechanism and has been proposed to explain why polygenic scores achieve higher predictive accuracy in higher BMI quantiles than in lower ones [51]. While intriguing, these deviations from our theory are relatively minor and determining the precise mechanism is beyond the scope of the current study.

Given the accuracy of our predictions, we can adjust for GWAS power by first computing $N^{\prime }$ for each binary trait and then scaling z-scores according to Equation 2. We estimated that $N^{\prime }$ is approximately 192K and 333K for the current schizophrenia and coronary artery disease GWAS, respectively (Methods, Supplementary note, Supplementary Table 3). Even after matching all three example traits in Figure 1 to the same $N^{\prime }$, we continue to see differences in the CDFs of |z|-scores and MAF for independent GWAS hits (Figure 3E). This more rigorous comparison shows that differences in GWAS power do not explain why brain-related differ from other traits in genetic architectures, suggesting that other biological or evolutionary mechanisms are involved.

### Brain-related traits have large mutational target sizes and relevant variants are under strong selection

**Figure 4:**
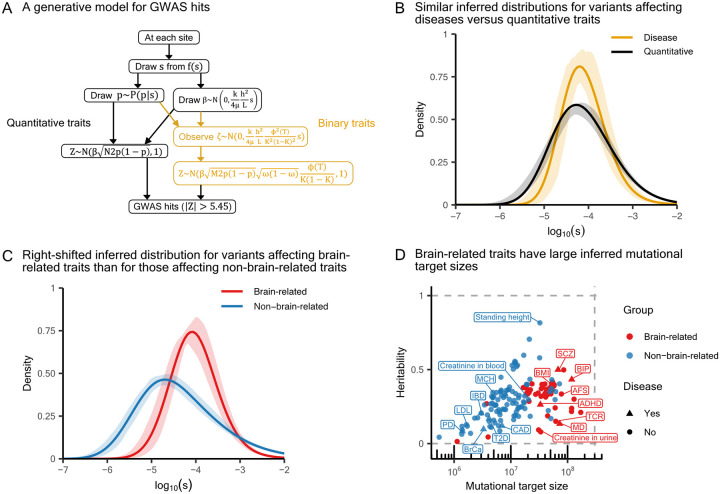
Stronger selection on brain-related variants and larger mutational target sizes for brain-related traits. **A)** The model that generates MAF, effect size, and observed GWAS z-score at each genomic position, adapted from [15] to include binary traits. μ is the mutation rate, and k≡4μ/E[2q(1-q)s]≈1 depends on f(s) and demography (see Supplementary Section 1.5 in [15]). $h^{2}$ for a binary trait refers to heritability on the liability scale. **B)** Inferred distributions of selection coefficients estimated from 151 quantitative traits and 13 human complex diseases, respectively, with 90% confidence envelopes shown. **C)** Inferred distributions of selection coefficients when trait were instead grouped into 54 brain-related traits and 110 non-brain-related traits, with 90% confidence envelopes shown. **D)** Median estimates of heritability and mutational target size, inferred jointly with the respective f(s) shown in panel C. The gray lines are drawn at $x=3\times 10^{8}$, and y=0 and 1. MCH: Mean corpuscular haemoglobin.

To formally investigate alternative explanations, we turned to the generative model for GWAS hits of a quantitative trait previously developed by Simons *et al*. [15]. We extended this model to accommodate binary traits using Equation 1 (Figure 4A). In this framing, mutations affecting the trait arise at a rate that depends on the trait’s mutational target size L, and are then assigned a selection coefficient s>0 from a distribution f(s). The frequency of the mutation at the time the GWAS is performed, p, is sampled from a distribution that arises from under-dominant selection with the selection coefficient s and the effects of genetic drift during the demographic history of the population. We assume a demographic model estimated for the British population [52] and a mutation rate of 1.25 × 10^−8^ [14]. The effect size of the mutation, β, also depends on s: specifically, β∣s~N(0,c⋅s), where c is a trait-specific constant that depends on the heritability per site in the target, $h^{2}/L$. The variant is then assigned a z-score sampled from a distribution that depends on its effect size, allele frequency, and GWAS sample size. When this z-score exceeds the genome-wide significance threshold, the variant is considered a GWAS hit. Below, we fit f(s) using a spline function with four knots (see Supplementary Section 4.8 in [15]), thus our full model includes six parameters per trait: four for f(s), as well as $h^{2}/L$, and L.

First, we fit the extended Simons model to six complex diseases that have more than 100 approximately independent hits (trait-specific f(s) shown in Figure S20), as previous work suggests the inference of evolutionary parameters should be well powered in this case [15]. Two of the six diseases, schizophrenia and major depression, are brain-related. For comparison, we considered alternative models: fitting effect sizes with a normal distribution, and the α-model [21], which imposes a negative relationship between effect sizes and allele frequencies.

We quantified the goodness-of-fit of the different models by computing a residual p-value, defined previously as Pr $\left(z\right.>\left|z_{i}\right||\left|z_{i}\right|>5.45,p_{i}$, model) [15]. If we correctly model the distribution of z-scores among GWAS hits, then these residual p-values should be uniformly distributed between 0 and 1. To avoid overfitting, we split the genome into approximately independent blocks [53], each time inferring the model on 90% of the blocks and computing residual p-values for the held-out 10%. Across diseases, our pleiotropic stabilizing selection model provides a better fit to the residual p-values than the two simpler heuristic models (Figure S21). We next conducted Kolmogorov-Smirnov tests to determine whether the overall distribution of residual p-values for each trait matches the expected uniform distribution. At a false discovery rate of 0.05, we find that the simpler heuristic models provide a poor fit to the data; notably, we can reject the normal model for 5 of the 6 diseases, the α-model for 4, and the pleiotropic stabilizing selection model for none (Supplementary Table 4). These results confirm that the extended Simons model provides a robust fit for complex diseases and support the view that common variants affecting complex diseases are shaped by pleiotropic stabilizing selection [7, 33, 34].

To include traits with fewer hits in our inference, the Simons model further allows grouping traits to estimate a shared f(s) distribution, thereby reducing the number of trait-specific parameters from six to two — namely, $h^{2}/L$ and L. We therefore expanded our analysis to include all 151 quantitative traits and 13 diseases, each with more than 20 approximately independent hits. When stratifying traits into complex diseases and quantitative traits, the inferred distributions of selection coefficients have similar modes (Figure 4B).

By contrast, for traits with more than a hundred GWAS hits, the inferred trait-specific f(s) distributions differ markedly between brain-related and other complex traits (Figure S22). These findings motivated us to group traits into brain-related and non–brain-related categories. The two shared f(s) distributions reflect the stronger selective constraint acting on mutations affecting brain-related traits (Figure 4C). Moreover, the shared f(s) for brain-related traits is narrower than that for non–brain-related traits, which suggests a narrower distribution of effect sizes for brain-related traits, consistent with previous studies (see Introduction and [31, 32]).

We also found that brain-related traits tend to have substantially greater mutational target sizes than other complex traits (Figures 4D and S23), echoing previous studies that found brain-related traits to be more highly polygenic than other traits (see Introduction and [25, 28–30]). To contextualize these estimates, approximately 10% of the genome has been estimated to be constrained by selection and is therefore considered putatively functional [54–56]. For brain-related traits, our median inferred mutational target size is approximately 1.32% of the genome, compared with 0.27% for non-brain-related traits.

In contrast to selection and mutational target size, heritability estimates do not differ significantly between brain-related and other traits (Figures 4D and S23). In turn, estimates of $h^{2}/L$, which is the key scaling parameter governing GWAS power and relating trait architectures within each category [15], are markedly lower for brain-related traits.

To validate our heritability estimates, we compared them with the recent whole-genome sequencing–based estimates ($h_{\text{WGS}}^{2}$) [57]. For the set of overlapping quantitative traits, our $h^{2}$ estimates derived from brain-related and non-brain-related f(s) distributions show greater concordance with $h_{WGS}^{2}$ than estimates obtained under a single quantitative f(s) model, suggesting that grouping traits by CNS status improves model performance (Figure S24).

### Strong selection and large mutational target size explain the distinct architecture of brain-related traits

We next wanted to see if the inferred larger mutational target sizes and stronger selection on variants could explain the genetic architectures of brain-related traits. In particular, we used simulations to see the impact of mutational target size and selection on the MAF and |z|-score distribution of GWAS hits.

**Figure 5:**
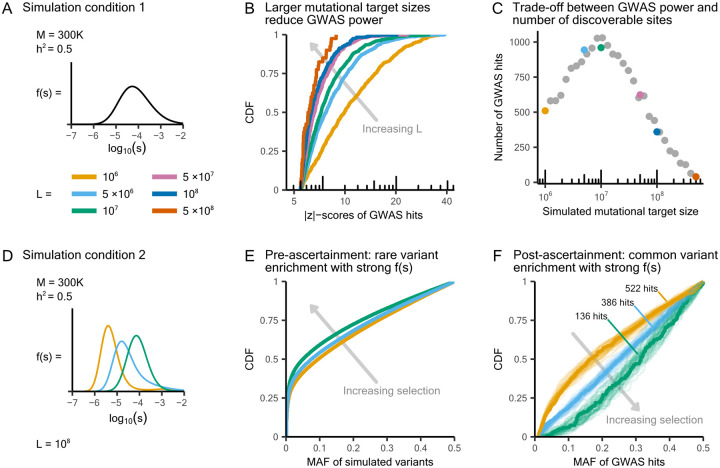
Recovering the genetic architectures of brain-related traits through simulations. **A)** Simulation model for traits with varying mutational target sizes. We fixed f(s) to the shared distribution inferred from 151 quantitative traits as shown in Figure 4B. Colors in panels B and C correspond to the simulation settings shown in A. **B)** Larger L values lead to narrower distributions of GWAS hit |z|-scores. **C)** Nonlinear relationship between L and GWAS hit count. We ran additional simulations at finer increments of L and plotted them as gray dots. **D**) Simulation model for traits with varying distributions of selection coefficients. Colors in panels E and F correspond to the simulation settings shown in D. **E)** In the absence of GWAS ascertainment, variants simulated under stronger f(s) distributions tend to have lower MAF. **F)** After GWAS ascertainment (MAF > 1% and |Z|>5.45), stronger f(s) simulations yield GWAS hits enriched for higher MAF. For each f(s) distribution, we ran 50 simulations, highlighted the median CDF curve of the MAF of simulated hits, and labeled the corresponding number of hits.

Under a fixed f(s) distribution — e.g., the one estimated from 151 quantitative traits — we simulated a trait with sample size 300K and trait heritability 0.5, varying only the mutational target size (Figure 5A). We found that larger values of L lead to steeper CDF curves for GWAS hit |z|-scores (Figure 5B). This occurs because GWAS power is linearly proportional to the scaling parameter $h^{2}/L$ (Figure 4A). The number of GWAS hits, on the other hand, scales nonlinearly with L (Figure 5C). While increasing L makes more genomic sites relevant to the trait [15], the power to discover each individual site declines. These opposing effects interact to produce the observed nonlinear trend. Nevertheless, the MAF distributions of GWAS hits are largely insensitive to changes in mutational target size, indicating that mutational target size variation cannot capture the observed enrichment of higher-MAF hits in brain-related traits (Figure S25).

We next sought to understand the impact of selection by holding the mutational target size at 10^8^ bp and varying the distribution of selection coefficients in our simulations (Figure 5D). In the absence of GWAS ascertainment, variants simulated under stronger f(s) distributions are generally rarer than those from weaker f(s), consistent with intuition (Figure 5E). Intriguingly, GWAS ascertainment — that is, restricting to common variants and selecting hits that surpass the genome-wide significance threshold — reverses this MAF relationship (Figure 5F). When trait-affecting variants are under stronger selection, only small effect variants can reach sufficiently high frequencies to be included in GWAS. Due to this ascertainment bias, an over-representation of small effect variants in turn leads to an enrichment of variants with higher MAF (Figure S26B). Additionally, stronger f(s) distributions also result in steeper |z|-score distributions and fewer hits (Figures S26 C to D).

### Rare variant burden tests suggest strong selection on brain-relevant genes

Although simulations show that a stronger f(s) distribution generates GWAS hits of higher MAF and lower |z|-scores — patterns consistent with what we observed for brain-related traits — these same GWAS hits are also the signals we used to infer f(s). To seek orthogonal evidence in support of stronger selective constraint on genetic variation influencing brain-related traits, we next turned to a genic perspective: are genes relevant to brain-related traits also under stronger selection compared to genes relevant to non-brain-related traits?

To that end, we leveraged previously reported estimates of the fitness effects of loss-of-function (LoF) variants, denoted $s_{\text{het}}$ [58]. These estimates quantify selection against heterozygotes and serve as a metric of gene-level constraint. Moreover, from burden tests of these LoF variants, we obtained gene-to-trait effect estimates, $\hat{\gamma }$, which we then used to derive unbiased estimates of the squared effects, $\hat{\gamma^{2}}$ (Methods). One caveat to note is that our analysis did not include burden tests on binary traits, primarily due to limited data and the fact that statistical power is biased toward detecting risk-increasing effects in the rare variant regime.

Under a model of pleiotropic stabilizing selection, in which selection against LoF variants is mediated through their effects on traits, Spence *et al*. recently showed that on average $\hat{\gamma^{2}}$ increases with increasing $s_{\text{het}}$ across a set of independent quantitative traits [39]. In addition, multiple regression analyses showed that across 17 tissues, whether or not a gene is expressed in the “frontal cortex” is among the strongest predictors of estimated gene constraint, $s_{\text{het}}$ (Figure S27). Together, these observations led to the hypothesis that brain-relevant genes are more constrained than other genes and that in constrained genes, the fitness effects of LoF variants should correlate more strongly with their effect on brain-related traits than on non-brain-related traits.

**Figure 6:**
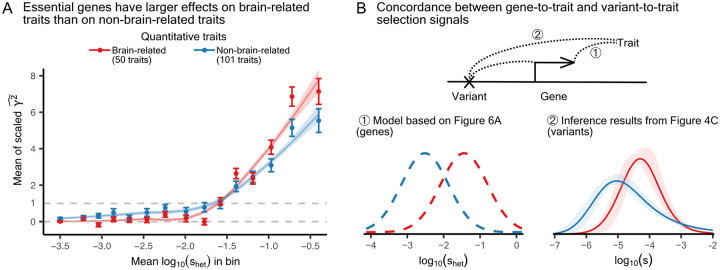
Burden tests provide aligned evidence of stronger selection on CNS-relevant genes. **A)** Mean squared genic effects on brain-related versus non-brain-related quantitative traits, with genes grouped into 15 quantiles of $s_{\text{het}}$ estimates [58]. The estimated $\hat{\gamma^{2}}$ values are scaled so that the mean across all gene-trait pairs in the tenth bin equals one. All estimates are shown with a 95% confidence interval computed from the standard error. We bootstrapped genes at the trait level and showed the mean and 95% confidence intervals of the fitted LOESS curves. **B)** Comparing selection signals from gene-to-trait and variant-to-trait associations. ① Brain-relevant genes have a stronger distribution of constraint values than genes relevant to other traits. The two curves are hypothetical and are intended to illustrate the relationship between their modes. ② Brain-relevant variants have a stronger distribution of selection coefficients than variants relevant to other traits.

Binning genes by $s_{het}$, we indeed found that genes that are more constrained have larger relative squared effect sizes, $\hat{\gamma^{2}}$, on brain-related than on non-brain-related quantitative traits, and vice versa for less constrained genes (Figure 6A). If these two trait categories had systematic differences in heritability or other factors, one curve would be consistently higher across the range. Instead, the two curves switch order, ruling out this possibility and supporting the hypothesis that effects on brain-related traits more strongly correlate with fitness consequences. In addition, we pruned the set of quantitative traits to 32 approximately uncorrelated traits, comprising 11 brain-related traits and 21 non-brain-related traits (Methods, Figure S28). Although the signal is noisier, we observed the same S-shaped trend (Figure S29).

Figure 6A suggests a model in which brain-relevant genes have a stronger distribution of constraint values than genes relevant to non-brain-related traits (① of Figure 6B). If selection is stronger on changes in gene activity in the CNS relative to other tissues, this could help explain why we inferred a stronger distribution of selection coefficients for brain-relevant variants compared to variants relevant to other traits (Figure 4C, plotted again in ② of Figure 6B). Therefore, the strong selection signals we found for the brain-related traits are concordant at both the variant and gene levels.

## 3 Discussion

What are the processes that shape the genetic architecture of complex traits? Do certain groups of traits share common features, and if so, what causes them to collectively differ from other trait categories?

We found that brain-related quantitative traits and complex diseases share distinct genetic architectures, in that their GWAS hits have modest significance, narrow effect sizes, and are enriched at higher MAF. These features can be explained by the combination of brain-related traits generally having larger mutational target sizes than other complex traits and, importantly, experiencing markedly stronger selection on the genetic variation that affects them.

Our variant-level analyses were based on common variants, which are largely regulatory [59]. If selection on variants affecting brain-related traits is stronger than for other traits, then we would expect a greater proportion of their heritability to come from rare non-coding variants. In line with these expectations, recent studies using whole-genome sequencing data have found that brain-related traits exhibit relatively strong heritability enrichment from rare variants and the highest enrichment from non-coding variants across all traits examined [57].

**Figure 7:**
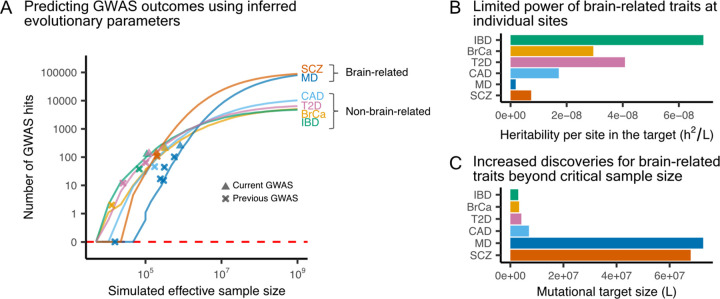
Explaining past and predicting future disease GWAS discoveries. **A)** Expected disease GWAS outcomes as a function of sample size, computed using the inferred f(s) for brain-related and non-brain-related traits (Figure 4C), along with their corresponding estimates of $h^{2}$ and L as shown in Figure 4D. We evaluated a range of sample sizes across 35 grid points and plotted the curves using the median values across 10 simulations at each point. Our inference was based on the current GWAS (see Supplementary Table 2). For reference, we overlaid the reported number of hits and sample sizes from selected earlier GWAS (IBD [73]; BrCa [74]; T2D [75, 76]; CAD [77]; MD [61, 63–66]; and SCZ [78]). **B)** Inferred $h^{2}/L$ estimates used in the simulations. **C)** Inferred L estimates used in the simulations.

As GWAS sample sizes continue to grow, we can use the inferred evolutionary parameters to explain past GWAS findings and predict future results. Focusing on the six complex diseases with more than a hundred GWAS hits, Figure 7A shows the simulated GWAS discoveries as a function of the effective study size (Equation 1). In particular, our findings for major depression recapitulate the historical trajectory: early studies showed a prolonged period with few or no significant findings [60–62], followed by a recent sharp increase in the number of detected loci [63–67]. Our inference provides a mechanistic explanation for these historical trends. To a good approximation, variants reach genome-wide significance when their contribution to phenotypic variance exceeds a threshold inversely proportional to the study’s sample size. Given the low heritability per site for major depression (Figure 7B), larger sample sizes are required to surpass this threshold, after which the number of discoveries sharply rises due to its large mutational target size (Figure 7C). More generally, we expect future GWAS discoveries for brain-related traits to increase more rapidly than those for non-brain-related traits, ultimately reaching higher saturation points.

Importantly, our findings should not be interpreted as evidence that brain-related traits, especially the behavioral-cognitive traits, are themselves under stronger selection. Selection on brain-related variants and genes does not necessarily operate through the traits we considered. Rather, it can be posited that selection acts primarily on core aspects of brain development and functioning, more strongly than on other tissues. In support of this view, among constrained genes depleted of missense and LoF variants, 30% are highly expressed in the brain, compared with only 14% in lymphocytes, which rank second across tissues [68]. However, these processes are difficult to define as traits, let alone to quantify or measure. The brain-related traits we analyzed therefore serve only as proxies, tagging the variants and genes involved in these essential biological processes.

Additionally, it is within the Simons modeling framework that our findings provide the most plausible explanation for the genetic architectures of brain-related traits. We also examined potential ways in which brain-related traits might violate the modeling assumptions. For example, since brain-related traits are highly correlated [69], they could in principle collapse onto a single underlying trait, thereby undermining the assumption of a high-dimensional trait space. However, simulations assuming no pleiotropic variant effects cannot recover the observed MAF distributions of GWAS hits for brain-related traits (Methods, Figure S30).

Why would selection on genetic variation be stronger for brain-related traits compared to traits mediated by other tissues? Notably, brain tissues are distinct in that expression quantitative trait loci (eQTL) effects show high levels of sharing within the brain, yet remain largely unshared with non-brain tissues [70]. This suggests that variants regulating gene expression in the brain may be largely brain-specific. With most common variants being regulatory, we can envision the strong selection happening in three ways, where comparing CNS to other tissues: i) variants could typically have greater effects on the activity (e.g., expression) of genes; ii) changes in the activity of genes could typically have greater effects on traits; and iii) changes in the activity of genes typically affect more selected traits. The genic analyses we presented (Figure 6) suggest that changes to gene expression involve greater fitness effects in the CNS than in other tissues, which in turn supports the latter two models (without excluding the first).

Furthermore, our analysis suggests that traits that are mediated by the same tissues tend to have similar architectures and thus similar evolutionary parameters. Brain-related traits appear to occupy a “corner” in the architecture space, which is why their genetic architectures are more clearly set apart from the architecture of traits mediated by other tissues, but we also see a clustering of architectures for cardiovascular and skeletal muscle traits, for example (Figure S9 and S10). The omnigenic model [71, 72] suggests why trait architectures might cluster by tissues. If many of the regulatory variants that affect gene activity in given cell types contribute to heritable variation in traits that are mediated by these cell types, then we would expect these traits to share similar mutational target sizes and distributions of selection effects; the sharing of effect size distributions (scaled appropriately) follows from the shared distribution of selection effects under the pleiotropic stabilizing selection model [14]. Nonetheless, our inference suggests that there is substantial variation in mutational target sizes and distribution of selection effects among brain-related traits (Figures S22 and S23). This too is to be expected: even setting aside the coarseness in associating traits with tissues and the uncertainty in our inferences, traits that are mediated by the same tissue are expected to have different numbers of “core genes” and to be differentially affected by different cellular circuits.

The case of body composition-related traits is especially interesting from this point of view. As noted above, these traits are mediated by a mixture of tissues, including the CNS (Figure S11). Their architectures and inferred evolutionary parameters appear to fall between those of brain-related traits mediated primarily by the CNS and those of non-brain-related traits (Figures 4D and S23). More generally, this suggests that the architecture of common variation underlying complex traits can be approximated as a superposition of architectures associated with tissues, with some trait-specific effects in each tissue.

## 4 Methods

### Identifying approximately independent hits using GCTA-COJO.

From GWAS summary statistics, we applied GCTA-COJO [38] to identify conditionally independent hits and re-estimated variant effect sizes $\overset{ˆ}{\beta }$ on each trait. We used parameters -cojo-p 5e-8 and -cojo-slct, along with a LD reference panel consisting of genotypes from a subset of 10,000 White British individuals from the UK Biobank. We removed hits with a minor allele frequency (MAF) below 1%, those located in the HLA region, and those with INFO scores below 0.8.

### Data on 151 quantitative traits.

#### GWAS summary statistics.

We downloaded GWAS summary statistics for 305 quantitative phenotypes (Supplementary Table 1; same trait list as in [39]) from the Neale Lab (http://www.nealelab.is/uk-biobank, version 3). Specifically, these results were based on 361,194 individuals of White British ancestry in the UK Biobank. All the phenotypes were rank-based inverse normal transformed. Age, age^2^, inferred sex, age × inferred sex, age^2^ × inferred sex, and principal components 1 to 20 were included as covariates.

#### SNP heritability.

Estimates for the 305 quantitative traits were downloaded from the Neale Lab.

#### Number of significant GWAS hits.

We followed the same procedure as described in the subsection for identifying approximately independent hits.

For this study, we focused on 151 quantitative traits, each with at least 20 significant hits and a SNP $h^{2}$ greater than 5% (Supplementary Table 1).

### Data on 13 complex diseases.

#### GWAS summary statistics.

Since the UK Biobank cohort has been found to be healthier than the general population [79], we turned to publicly available latest GWAS summary statistics from case-control studies. We restricted our analyses to studies on cohorts of European ancestry, as the demographic model in [15] was specifically derived for the European population. For several diseases, the complete results require additional approval from 23andMe. We thus used only the publicly available version, excluding the 23andMe cohort.

#### Number of significant GWAS hits.

We followed the same procedure as described in the subsection for identifying approximately independent hits.

Using the same cut-off of more than 20 genome-wide independent hits, we curated a list of 13 complex diseases (Supplementary Table 2). Although not exhaustive, this list defines the scope of this present study.

### Stratified LD score regression (S-LDSC)

GWAS summary statistics on all 164 traits were processed by the “munge_sumstats.py” script provided by LDSC developers (https://github.com/bulik/ldsc, v1.0.1) [80]. LD scores were computed based on European ancestry participants in the 1000 Genomes Phase 3 dataset [81]. To identify key cell types for each trait, we conducted cell type-specific analyses with S-LDSC [26]. Finucane et al. categorized 220 distinct cell types into 10 categories: *adrenal / pancreas, cardiovascular, central nervous system (CNS), connective / bone, gastrointestinal, immune / hematopoietic, kidney, liver, other, and skeletal muscle*. Each cell-type-specific annotation was then added to the baseline LD model (v1.2) to evaluate heritability enrichment. The resulting regression coefficient z-scores were converted to one-sided p-values, shown in Figures 2B and S4 to S6. To account for multiple testing, we applied a Bonferroni correction, setting the significance threshold to a p-value of 0.05/(220 × 164).

In Figures 2D, S9, and S10, the p-values for each cell-type category is obtained from meta-analyses using the aggregated Cauchy association test (ACAT), which is robust to the correlations among the constituent p-values [41]. Specifically, within each category, p-values were transformed into Cauchy variables and aggregated through $T=\sum_{i=1}^{k}\frac{1}{k}\text{tan}\left\{\left(0.5-p_{i}\right)\pi \right\}$. The resulting p-value can be obtained from the Cauchy distribution with 0.5-{arctan(T)}/π.

### Thresholding and downsampling quantitative traits.

For a given quantitative trait, we obtained recorded trait values from approximately 360K unrelated White British individuals in the UK Biobank. We first conducted GWAS on rank-based inverse normal transformed phenotypes using PLINK 2.0 [82], with parameters -glm hide-covar and -covar-variance-standardize. Age, age^2^, sex, age × sex, array, center and principal components 1 to 20 were included as covariates in the regression model. We then identified conditionally independent hits using GCTA-COJO as described above.

For the thresholding analysis, we dichotomized the continuous trait into ten binary traits (cases=2 and controls=1 following PLINK format), representing a disease prevalence of 1%, 2%, 5%, 10% and 20% on either the lower or the upper tail of the phenotypic distribution. To reduce computational burden, we ran case-control GWAS only on the list of previously identified hits with flag -extract and modified parameter -glm firth-fallback hide-covar.

For the downsampling analysis, we drew five subsamples, each with sample sizes calculated to match with the prevalence rates of 20%, 10%, 5%, 2% and 1% used to create previous binary traits (Supplementary notes). Smaller subsets were ensured to nest within larger ones. Phenotypes in each subset were again rank-based inverse normal transformed to run GWAS using the same variant list filter.

Lastly, z-scores from each of the ten thresholded or five downsampled GWAS were linearly regressed on z-scores from the original GWAS.

These procedures were repeated for six medically relevant traits, as they are commonly used in clinical settings to declare disease status based on specific cutoffs. These traits include LDL, BMI, hemoglobin A1c (HbA1c), diastolic blood pressure, systolic blood pressure, and heel bone mineral density T-score. The corresponding UK Biobank data fields are 30780, 21001, 30750, 4079, 4080, and 78, respectively.

### Estimating $N^{\prime }$ for binary traits and re-scaling GWAS hit z-scores.

For a given complex disease, we defined in Eq. 1 that $N^{\prime }\approx M\omega (1-\omega )\frac{\phi (T{)}^{2}}{K^{2}(1-K{)}^{2}}$ is the sample size needed on the underlying liability scale to reach equivalent power as of the original case-control study. Let $p_{i}$ be the MAF of SNP i and ${\hat{\zeta }}_{i}$ be its estimated effect size on the log odds ratio scale. Given that ${\hat{\zeta }}_{i}\sim 𝒩\left(\zeta_{i},\frac{1}{2Mp_{i}\left(1-p_{i}\right)\omega (1-\omega )}\right)$ (Supplementary notes), we estimated $\hat{M\omega (1-\omega }\omega )$ with the median value of $\frac{1}{2p_{i}\left(1-p_{i}\right)SE^{2}\left(\zeta_{i}\right)}$ across all SNPs in the summary statistics file. Disease prevalence values, K, were taken from the GWAS source paper when available. Otherwise, we used prevalence estimates in the European population. ϕ(T) is the density at the liability threshold, assuming that the underlying liability follows a standard normal distribution.

When standardizing two traits to have comparable sample sizes, regardless of whether the trait is quantitative with reported sample size N or binary with estimated sample size $N^{\prime }$, we simply scale the GWAS hit z-scores of the trait with larger sample size with $\sqrt{\frac{N_{\text{small}}}{N_{\text{big}}}}$.

### Inferring $f(s),h^{2},\text{and}\;L$; and simulating GWAS hits.

We used the scripts developed by Simons *et al*. for analyzing quantitative traits [15]. The complete inference framework is described in detail in their Supplement. Here, we outline the relevant key concepts. With the significant GWAS hits for a given set of traits, we used a maximum likelihood approach to jointly infer a shared f(s) across traits, along with trait-specific parameters, $h^{2}$ and L. The log likelihood of observing a GWAS hit i is

$$ℒ{ℒ}_{\text{hit}\;i}=\text{log}\left(\frac{P\left(Z_{i},p_{i}|h^{2},L,f\left(s\right)\right)}{P\left(\text{hit}|h^{2},L,f\left(s\right)\right)}\right).$$

By the chain rule in probability, the numerator is $P\left(Z_{i}|p_{i},h^{2},L,s\right)P\left(p_{i}|s\right)$. The site frequency spectra, $P\left(p_{i}|s\right)$, are estimated with forward simulations assuming a European demographic model. The z-scores then follow $𝒩\left(0,D\cdot 2p_{i}\left(1-p_{i}\right)N\frac{h^{2}}{L}s+1\right)$ up to a constant term D. The denominator accounts for GWAS ascertainment and is a double integral of the numerator for |z|>5.45 and p>1%. Details can be found in Supplementary Section 3.3 of [15].

For a given f(s) distribution, we first find each trait j’s maximizing $\hat{h^{2}}$ and $\hat{L}$ for

$$ℒ{ℒ}_{\text{trait}\;j}=\left(\sum_{i}ℒ{ℒ}_{\text{hit}\;i}\right)-0.05\left(\frac{\hat{L}}{3\cdot 10^{8}}+\frac{\hat{h^{2}}}{1}\right),$$

where the minus term penalizes estimates beyond reasonable thresholds. The shared f(s) across traits then maximizes $\left(\sum_{j}ℒ{ℒ}_{\text{trait}j}\right)$. Confidence intervals are obtained by bootstrapping and resampling genomic blocks. Details can be found in Supplementary Sections 2.8, 3.5, and 3.7 to 3.9 of [15].

To extend the inference framework to accommodate binary traits, we simply transferred effect sizes from log odds ratio scale to the liability scale with Eq. 6. For binary traits, $h^{2}$ denotes heritability on the liability scale, and N represents the corresponding effective sample size on that scale, $N^{\prime }$.

We further adopted the scripts provided by Simons *et al*. [15] to simulate GWAS hits of a trait, with input parameters $f(s),h^{2}$, and L. Briefly, we simulated L variants with MAF and z-scores drawn from $P\left(p_{i}|s\right)$ and $P\left(Z_{i}|p_{i},h^{2},L,s\right)$ and outputted the variants with $p_{i}>1\%$ and |Z|>5.45. Details can be found in Supplementary Section 5.1 of [15].

To modify the Simons model into a one-dimensional trait space model, i.e., assuming that variants do not have pleiotropic effects, we followed results in Simons et al. [14]. Contrary to a high-dimensional trait space in which $\beta \sim 𝒩\left(0,D\cdot \frac{h^{2}}{L}s\right)$, in a one-dimensional trait space β equals $\sqrt{D\cdot \frac{h^{2}}{L}s}$. It follows that the z-scores for a given variant should instead be drawn from $𝒩\left(\sqrt{D\cdot 2p_{i}\left(1-p_{i}\right)N\frac{h^{2}}{L}s},1\right)$.

### LoF burden tests on 151 quantitative traits.

Summary statistics for LoF burden tests on 112 quantitative traits were downloaded from Milind *et al*. [83]. For the remaining 39 quantitative traits, we followed the scripts they provided and conducted gene-based burden tests with REGENIE [84]. A whole genome regression model is fit in the first step to make Leave One Chromosome Out (LOCO) phenotypic predictions. We filtered variants for MAF > 1%, missingness < 10%, Hardy-Weinberg equilibrium test p-value < 10^−15^ and applied linkage disequilibrium pruning with 1000 variant windows, 100 variant shifts, and $r^{2}<0.9$. In the second step of REGENIE, LoF burden tests were performed with LOCO predictions included as an offset term. We filtered LoF variants to have MAF < 1% and misannotation probability (estimated in [58]) < 10%. At both stages, we applied rank-based inverse normal transformation to all phenotypes and included age, age^2^, sex, age × sex, genotyping PCs 1 to 15, rare variant PCs 1 to 15, and WES batch as covariates. With the estimated gene-to-trait burden effects, $\overset{ˆ}{\gamma },{\overset{ˆ}{\gamma }}^{2}-\text{SE}(\overset{ˆ}{\gamma }{)}^{2}$ is an unbiased estimator of mean squared genic effects $\gamma^{2}$ [39, 83].

### Distinct subsets of (approximately uncorrelated) brain-related and non-brain-related Traits.

From the set of 164 complex traits, 50 quantitative traits and 4 complex diseases with meta-analysis p-values for CNS enrichment passing the Bonferroni threshold of 0.05/(10 × 164) are classified as CNS traits. The remaining 101 quantitative traits and 9 complex diseases are classified as non-CNS traits (Supplementary Table 1).

To reduce redundancy in the trait list, we pruned the set of 151 quantitative traits to 32 approximately uncorrelated traits as shown in Figure S28. Specifically, traits were sorted in decreasing order of meta-analysis p-values for CNS enrichment. Starting with the trait that had the strongest enrichment for CNS, *time to complete round*, we iteratively added traits whose pairwise genetic correlations with all previously selected traits did not exceed 0.5. Genetic correlation estimates were obtained from the Neale Lab. We excluded 27 biomarker traits that did not have genetic correlation estimates. In the end, we selected 32 approximately uncorrelated traits, of which 11 are CNS traits (Supplementary Table 1).

## Supplementary Material

Supplement 1

1

## Data Availability

Codes used for this article are available at https://github.com/huishengz/cns-selection. Data and results are available at https://doi.org/10.5281/zenodo.19154957.
