# Supplementary material for: Genetic architectures of brain-related traits are shaped by strong selective constraints
Source: bioRxiv. 2026 Mar 25:2026.03.22.713538. Preprint. [Version 1] doi: 10.64898/2026.03.22.713538 (PMC13042030; doi:10.64898/2026.03.22.713538)
Supplement: 1 [file NIHPP2026.03.22.713538V1-supplement-1.pdf]

bioRxiv preprint doi: https://doi.org/10.64898/2026.03.22.713538; this version posted March 25, 2026. The copyright holder for this preprint (which was not certified by peer review) is the author/funder, who has granted bioRxiv a license to display the preprint in perpetuity. It is made available under aCC-BY 4.0 International license.

Supplementary Materials

Table of Contents

Supplementary Notes:

GWAS power. 32
Relating effect sizes. 32
Figure S1: Relating effect sizes with the liability threshold model. 33
Binarizing and downsampling a quantitative trrait. 33
Figure S2: Reduction in GWAS power after binarizing a quantitative trait. 34
Insights from the formula for estimating N'. 34
Figure S3: A larger sample size is needed on the liability scale when the disease is rarer. 35

Supplementary Tables:

Table S1: Details of quantitative traits included in this study. 36
Table S2: Details of complex diseases included in this study. 37
Table S3: Key parameters for complex diseases included in this study 37
Table S4: Model fit of the normal distribution, the alpha model, and the Simons model. 38

Supplementary Figures:

Figure S4: S-LDSC functional enrichment analyses on 151 quantitative traits (first half). 40
Figure S5: S-LDSC functional enrichment analyses on 151 quantitative traits (second half). 41
Figure S6: S-LDSC functional enrichment analyses on 13 human complex diseases. 42
Figure S7: Contrasting neurological diseases with 10 other human complex diseases. 42
Figure S8: Squared effect sizes of GWAS hits for brain-related versus other traits. 43
Figure S9: Parameterizing the genetic architectures of complex traits (first half). 44
Figure S10: Parameterizing the genetic architectures of complex traits (second half). 45
Figure S11: Contrasting body composition-related traits with other quantitative traits. 46
Figure S12: Contrasting two birth coordinate traits with 151 selected quantitative traits. 47
Figure S13: Contrasting urinary traits with serum creatinine and other quantitative traits. 48
Figure S14: LDL GWAS power loss after lower-tail binarization. 48
Figure S15: BMI GWAS power loss after binarization or downsampling. 49
Figure S16: HbA1c GWAS power loss after binarization or downsampling. 50
Figure S17: Diastolic blood pressure GWAS power loss after binarization or downsampling. 51
Figure S18: Systolic blood pressure GWAS power loss after binarization or downsampling. 52

bioRxiv preprint doi: https://doi.org/10.64898/2026.03.22.713538; this version posted March 25, 2026. The copyright holder for this preprint (which was not certified by peer review) is the author/funder, who has granted bioRxiv a license to display the preprint in perpetuity. It is made available under aCC-BY 4.0 International license.

Figure S19: Heel BMD T-score GWAS power loss after binarization or downsampling. 53

Figure S20: Trait-specific distributions of selection coefficients for six complex diseases. 54

Figure S21: QQ plots of residual p-values for six complex diseases. 55

Figure S22: Comparing trait-specific $f(s)$ for brain-related versus non-brain-related traits. . . . 56

Figure S23: Histograms of inferred evolutionary paramters. 57

Figure S24: Comparison of heritability estimates obtained in this study with recently reported whole-genome sequence-based heritability estimates. 58

Figure S25: Variation in MAF distributions of GWAS hits cannot be recovered by changing L. 58

Figure S26: Impact of GWAS ascertainment on simulations under different $f(s)$ distributions. 59

Figure S27: Whether or not a gene is expressed in the brain strongly predicts s_{het}. 60

Figure S28: Heatmap of pairwise genetic correlations across 32 independent traits. 61

Figure S29: Mean squared genic effects on independent brain-related versus non-brain-related traits. 62

Figure S30: Assuming no pleiotropy for variant effects fails to recapitulate the observed variation in MAF distributions of GWAS hits. 62

bioRxiv preprint doi: https://doi.org/10.64898/2026.03.22.713538; this version posted March 25, 2026. The copyright holder for this preprint (which was not certified by peer review) is the author/funder, who has granted bioRxiv a license to display the preprint in perpetuity. It is made available under aCC-BY 4.0 International license.

Supplementary Notes

GWAS power.

Quantitative trait GWAS power. In a linear regression model of the form $Y \sim X\beta + \epsilon$, the estimated effect sizes are approximately distributed as $\hat{\beta} \sim \mathcal{N}(\beta, SE^2)$ [85]. For a quantitative trait GWAS, Y is a vector of standardized phenotype values, X is a vector of genotype values at a focal SNP, β is the additive effect size per copy of the effect allele, and the residual term $\epsilon \sim N(0, \sigma^2)$ captures the background genetic and environmental noise. If H_1 is true, i.e., $\beta \neq 0$, Z^2 approximately follows a non-central chi-square distribution, $Z^2 \sim \chi_1^2((\frac{\beta}{SE})^2)$. GWAS power is determined by this non-centrality parameter $(\frac{\beta}{SE})^2$. Furthermore, if N is the total number of individuals in the study, and X_i is centered so that $\bar{X} = 0$,

$$SE^2 \approx Var(\hat{\beta}) = \frac{\sigma^2}{NVar(X)} = \frac{\sigma^2}{2Np(1-p)},$$

where the last step assumes Hardy-Weinberg equilibrium at this locus. Assuming σ^2 is approximately the phenotypic variance, V_p, and the trait is standardized to have unit variance, we get

$$\text{quantitative trait GWAS power} \propto f\left(2Np(1-p)\beta^2\right). \tag{3}$$

Binary trait GWAS power. A case-control GWAS typically assumes a logistic regression model of the form

$$\ln(\frac{P}{1-P}) \sim \zeta X + \epsilon, \tag{4}$$

where $P = Prob(Y = 1)$, and ζ is the effect size of allele 1 measured on the log odds ratio scale.

	Case	Control
Risk allele count	a	b
Non-risk allele count	c	d

Table 1: Contingency table.

Given a contingency table as shown in Table 1 and assuming that all cell counts are non-zero,

$$\hat{\zeta} \sim N(\zeta, \frac{1}{a} + \frac{1}{b} + \frac{1}{c} + \frac{1}{d}),$$

approximately. Under the null hypothesis that $\zeta = 0$, we have $E(a) = 2M\omega p$ given that M is the number of cases plus the number of controls, and ω is the sample prevalence. Similarly, $E(b) = 2M(1-\omega)p$, $E(c) = 2M\omega(1-p)$, and $E(d) = 2M(1-\omega)(1-p)$. Plugging these expected values into the previous formula, it follows that $\hat{\zeta} \sim N(\zeta, \frac{1}{2Mp(1-p)\omega(1-\omega)})$. Therefore, we arrive at

$$\text{binary trait GWAS power} \propto f\left(2Mp(1-p)\zeta^2\omega(1-\omega)\right). \tag{5}$$

Relating effect sizes.

Note that the effect sizes obtained from a quantitative trait GWAS, β's, are measured in standardized units on an additive scale, whereas the effect sizes obtained from a binary trait GWAS, ζ's,

bioRxiv preprint doi: https://doi.org/10.64898/2026.03.22.713538; this version posted March 25, 2026. The copyright holder for this preprint (which was not certified by peer review) is the author/funder, who has granted bioRxiv a license to display the preprint in perpetuity. It is made available under aCC-BY 4.0 International license.

are measured on a log odds ratio scale. Previous studies found that if effect sizes are sufficiently small, then the relationship between these two effect sizes is

$$\beta \approx \frac{K(1-K)}{\phi(T)}\zeta, \tag{6}$$

where K is the prevalence of the disease in the population and $\phi(T)$ is the standard normal density at the liability threshold [11,86].

Here, we provide a derivation of this relationship. In a population carrying a variant with effect size β, the disease liability distribution is shifted to the right compared with a population without the variant (Figure S1). When the effect size, ζ, is sufficiently small, using Equation 4 we find that

$$\frac{K'}{1-K'} = \frac{K+\Delta K}{1-K-\Delta K} = \frac{K}{1-K}e^{\zeta} \approx \frac{K}{1-K}(1+\zeta),$$

which implies that

$$\Delta K \approx \frac{K(1-K)\zeta}{1+K\zeta} \approx K(1-K)\zeta.$$

In turn, when β is sufficiently small, the increase in prevalence can be approximated by

$$\Delta K \approx \beta \times \phi(T),$$

where $\phi(T)$ is the density at the liability threshold. The relationship between effect sizes on the liability scale and on the log odds ratio scale (Equation 6) follows from these expressions. When effect sizes are sufficiently large, this approximation breaks down, and ΔK depends on the shape of the liability distribution, which is typically assumed to be standard normal.

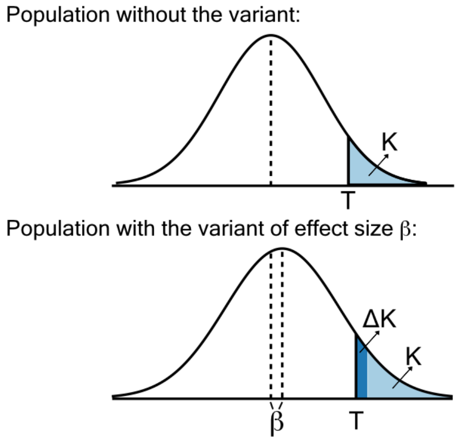

Figure S1: **Relating effect sizes with the liability threshold model.** Our derivation here assumes that effect sizes (both β and ζ) are small.

Binarizing and downsampling a quantitative trait.

Plugging Equation 6 into Equation 5, we can re-write binary trait GWAS power as

$$\text{binary trait GWAS power} \propto f\left(2Mp(1-p)\beta^2\omega(1-\omega)\frac{\phi(T)^2}{K^2(1-K)^2}\right). \tag{7}$$

bioRxiv preprint doi: https://doi.org/10.64898/2026.03.22.713538; this version posted March 25, 2026. The copyright holder for this preprint (which was not certified by peer review) is the author/funder, who has granted bioRxiv a license to display the preprint in perpetuity. It is made available under aCC-BY 4.0 International license.

To better understand how to relate the GWAS power of a quantitative trait and a binary trait, we next binarized a quantitative trait and ran a case-control GWAS. In the simplest case, we sampled the full set of cases and controls after binarization, that is, $N = M$ and $K = \omega$. According to Equation 7 and Equation 3, we see that binarizing a quantitative trait reduces GWAS power, with the case-control GWAS retaining $\frac{\phi(T)^2}{K(1-K)} * 100\%$ of the original GWAS power (Figure S2).

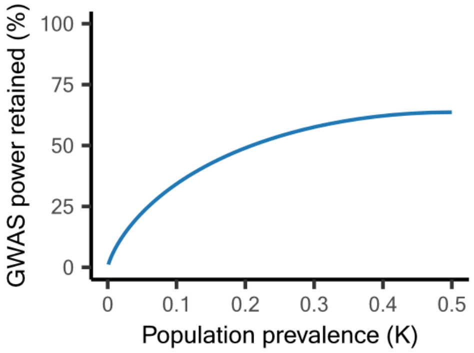

Figure S2: **Reduction in GWAS power after converting a quantitative trait to a binary trait.** We assume that the sample size remains the same and that the population prevalence matches the sample prevalence. $\phi(T)$ denotes the density at the liablity threshold, assuming liability follows a standard normal distribution.

Furthermore, the loss in GWAS power after trait binarization can be replicated by down-sampling the original trait to a new sample size N', given by Equation 1. The obtained Z-scores after binarization and downsampling both will be deflated by a factor of

$$Z_{after} = \sqrt{\frac{N'}{N}} Z_{before} = \sqrt{\frac{M\omega(1-\omega)\frac{\phi(T)^2}{K^2(1-K)^2}}{N}} Z_{before}. \tag{8}$$

Equation 8 gives the predicted slopes, shown as solid colorful lines in Figures 2C and D.

Insights from the formula for estimating N'.

The formula we derived for transferring the sample size of a case-control GWAS to the effective sample size on the liability scale, $N' \approx M\omega(1-\omega)\frac{\phi(T)^2}{K^2(1-K)^2}$, can be decomposed into two parts. From the first factor, $\omega(1-\omega)$, it is evident that the power of a case-control GWAS is maximized when the sample prevalence is 50%. The second factor, $\frac{\phi(T)^2}{K^2(1-K)^2}$, is the main reason why the estimated N' for the current schizophrenia GWAS, 192,273, is larger than its original sample size of M = 130,644 (Methods, Supplementary Table 3). For a given case-control study, i.e., when $M\omega(1-\omega)$ is fixed, a higher statistical power is achieved when the disease is less prevalent (Figure S3). One can also regard this as rewarding the extra data collection effort to recruit the same number of cases for a rarer disease.

Combining these elements, we arrived at a strategy for optimizing GWAS design. Suppose we want to conduct a GWAS on a quantitative trait, but with a constraint on budget. In other words,

bioRxiv preprint doi: https://doi.org/10.64898/2026.03.22.713538; this version posted March 25, 2026. The copyright holder for this preprint (which was not certified by peer review) is the author/funder, who has granted bioRxiv a license to display the preprint in perpetuity. It is made available under aCC-BY 4.0 International license.

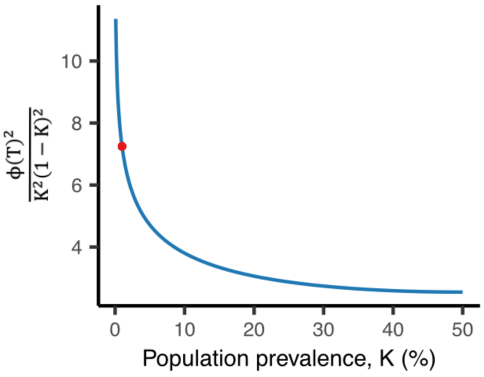

Figure S3: **A larger sample size is needed on the liability scale when the disease is rarer.** The red point is placed at $K = 1\%$, the assumed population prevalence for schizophrenia [37].

there is a limit on the number of individuals we can genotype or sequence. Under these circumstances, the optimal approach that maximizes GWAS power is to sample X individuals in the upper $K\%$ of the phenotypic distribution, match with an additional X individuals sampled from the lower $(1 - K)\%$ of the distribution, and conduct a case-control GWAS. As long as K is below 8.5%, the second factor will be larger than 4 (Figure S3), and therefore the resulting case-control GWAS will have greater statistical power than conducting a quantitative trait GWAS with $2X$ individuals.

bioRxiv preprint doi: https://doi.org/10.64898/2026.03.22.713538; this version posted March 25, 2026. The copyright holder for this preprint (which was not certified by peer review) is the author/funder, who has granted bioRxiv a license to display the preprint in perpetuity. It is made available under aCC-BY 4.0 International license.

Supplementary Tables.

Supplementary Table 1: **Details of quantitative traits included in this study.**

bioRxiv preprint doi: https://doi.org/10.64898/2026.03.22.713538; this version posted March 25, 2026. The copyright holder for this preprint (which was not certified by peer review) is the author/funder, who has granted bioRxiv a license to display the preprint in perpetuity. It is made available under aCC-BY 4.0 International license.

Supplementary Table 2: **Details of complex diseases included in this study.** Table of 13 complex diseases included in this study, with commonly used abbreviations, source of case-control GWAS summary statistics, and the number of genome-wide independent associations identified with GCTA-COJO [38] (Methods).

Disease	Abbreviation	GWAS source	Number of hits
Alzheimer's disease	AD	[87]	62
Attention deficit hyperactivity disorder	ADHD	[88]	24
Bipolar disorder	BIP	[89]	74
Breast cancer	BrCa	[90]	202
Coronary artery disease	CAD	[36]	217
Inflammatory bowel disease	IBD	[91]	127
Major depression	MD	[67]	249
Multiple sclerosis	MS	[92]	87
Parkinson's disease	PD	[93]	22
Prostate cancer	PrCa	[94]	134
Rheumatoid arthritis	RA	[95]	97
Schizophrenia	SCZ	[37]	196
Type 2 Diabetes	T2D	[96]	138

Supplementary Table 3: **Key parameters for complex diseases included in this study.** M: total sample size in the case-control study, including both cases and controls; ω: sample prevalence; K: population prevalence commonly used or estimated for the European population, taken from the GWAS source paper unless annotated with *; N': effective sample size required on the liability scale to reach equivalent power, calculated with Equation 1.

Disease	Reported M	Reported ω	$M\omega(1-\omega)$	$\widehat{M\omega(1-\omega)}$	K	N′
AD	487,511	17.6%	70,701	29,699	5% [97]*	140,016
ADHD	225,543	17.2%	32,121	22,449	5% [88]	105,835
BIP	840,309	7.1%	55,426	35,255	2% [89]	215,143
BrCa	247,173	54.0%	61,398	52,115	2% [98]*	318,030
CAD	1,165,690	15.6%	153,479	78,272	7% [36]	332,959
IBD	59,957	41.8%	14,586	12,836	0.5% [73]*	108,431
MD	2,000,702	20.6%	327,243	244,161	15% [67]	816,506
MS	115,803	41.0%	28,013	7,327	0.1% [92]	83,236
PD	482,730	7.0%	31,426	4,018	0.5% [93]	33,945
PrCa	140,254	56.4%	34,489	29,087	2% [98]*	177,504
RA	97,173	23.0%	17,209	8,159	0.5% [95]	68,921
SCZ	130,644	40.9%	31,579	26,529	1% [37]	192,273
T2D	659,316	9.5%	56,685	32,547	10% [96]	123,757

bioRxiv preprint doi: https://doi.org/10.64898/2026.03.22.713538; this version posted March 25, 2026. The copyright holder for this preprint (which was not certified by peer review) is the author/funder, who has granted bioRxiv a license to display the preprint in perpetuity. It is made available under aCC-BY 4.0 International license.

Supplementary Table 4: Evaluating model fit of the normal distribution, the alpha model, and the Simons model. For each trait-model combination, we conducted Kolmogorov–Smirnov (KS) tests to assess whether the residual p-values follow a uniform distribution. We then applied the Benjamini-Hochberg (BH) procedure to the KS p-values for each model to control the false discovery rate at 0.05.

	**Normal distribution**		**Alpha model**		**Trait-specific f(s)**	
**Disease**	**KS p-value**	**BH decision**	**KS p-value**	**BH decision**	**KS p-value**	**BH decision**
BrCa	9.521×10^{-10}	**Reject**	4.666×10^{-8}	**Reject**	0.641	Accept
CAD	1.756×10^{-12}	**Reject**	6.384×10^{-8}	**Reject**	0.242	Accept
IBD	3.992×10^{-3}	**Reject**	0.201	Accept	0.904	Accept
MD	0.765	Accept	0.764	Accept	0.538	Accept
SCZ	6.797×10^{-3}	**Reject**	0.0164	**Reject**	0.0498	Accept
T2D	5.133×10^{-9}	**Reject**	1.405×10^{-9}	**Reject**	0.191	Accept

bioRxiv preprint doi: https://doi.org/10.64898/2026.03.22.713538; this version posted March 25, 2026. The copyright holder for this preprint (which was not certified by peer review) is the author/funder, who has granted bioRxiv a license to display the preprint in perpetuity. It is made available under aCC-BY 4.0 International license.

Supplementary Figures.

bioRxiv preprint doi: https://doi.org/10.64898/2026.03.22.713538; this version posted March 25, 2026. The copyright holder for this preprint (which was not certified by peer review) is the author/funder, who has granted bioRxiv a license to display the preprint in perpetuity. It is made available under aCC-BY 4.0 International license.

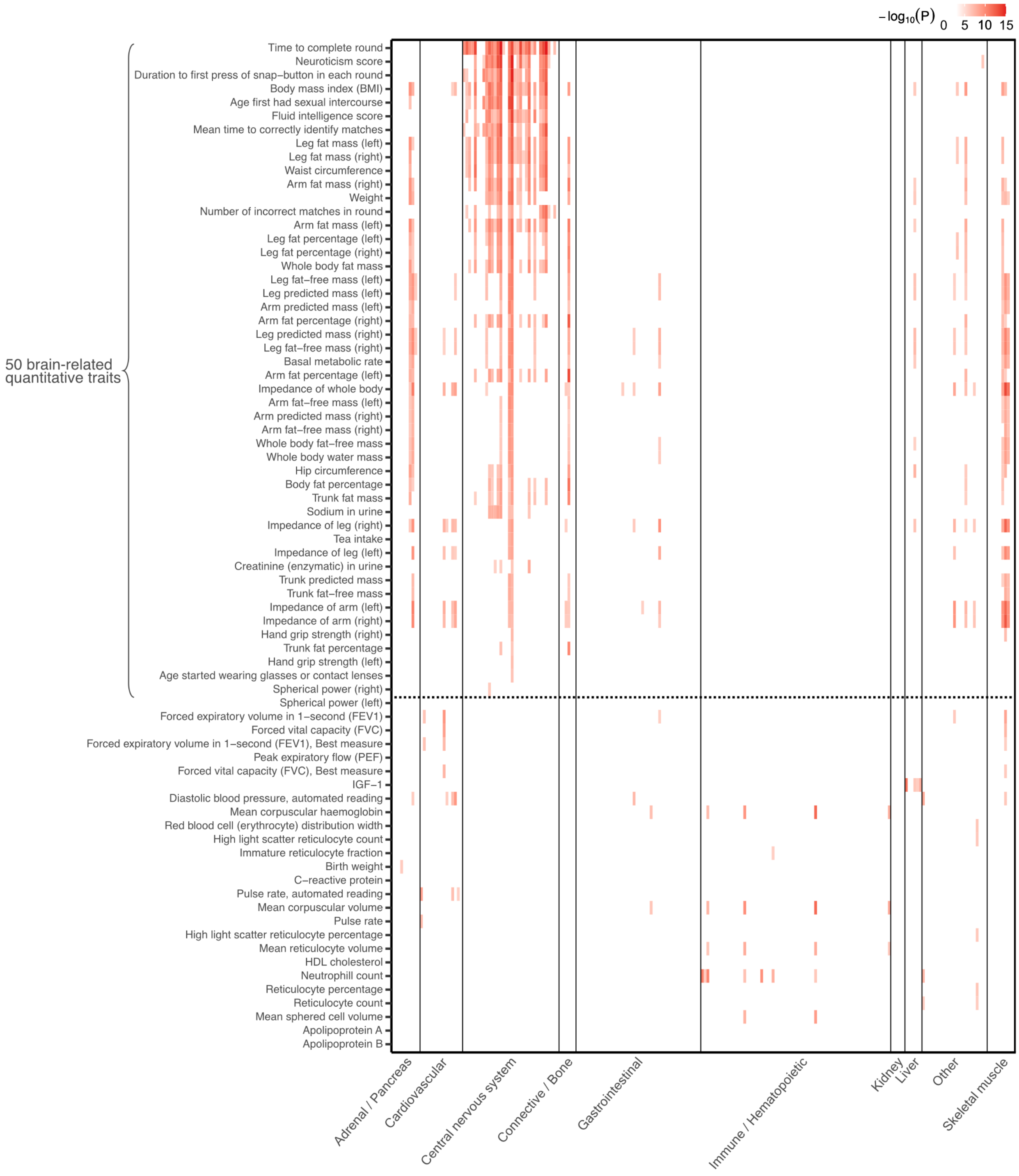

Figure S4: **S-LDSC functional enrichment analyses on 151 quantitative traits (first half).**
Each row consists of 220 cell types from 10 categories, colored by $-log_{10}(p)$ *for the coefficient* τ *in the S-LDSC model [26,27] if* $-log_{10}(p)$ *passes the Bonferroni-corrected threshold* $-log_{10}(0.05/(220 \times 164))$*. Traits are ordered by decreasing significance of the meta-analyzed p-values for CNS enrichment (Methods). The top 50 traits pass the significance threshold of* $-log_{10}(0.05/(10 \times 164))$ *and are therefore classified as brain-related.*

bioRxiv preprint doi: https://doi.org/10.64898/2026.03.22.713538; this version posted March 25, 2026. The copyright holder for this preprint (which was not certified by peer review) is the author/funder, who has granted bioRxiv a license to display the preprint in perpetuity. It is made available under aCC-BY 4.0 International license.

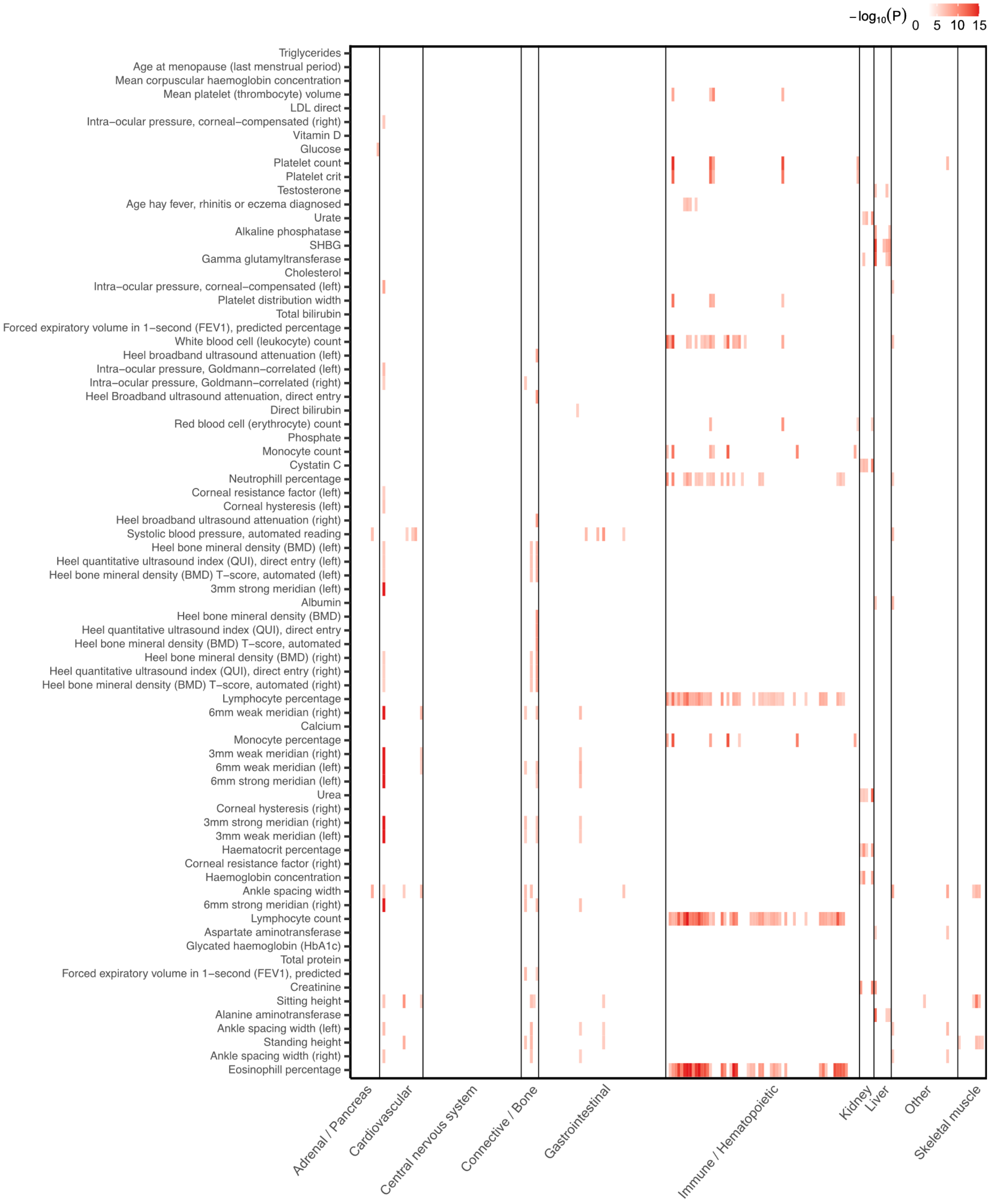

Figure S5: **S-LDSC functional enrichment analyses on 151 quantitative traits (second half).** *Each row consists of 220 cell types from 10 categories, colored by* $-log_{10}(p)$ *for the coefficient* τ *in the S-LDSC model [26, 27] if* $-log_{10}(p)$ *passes the Bonferroni-corrected threshold* $-log_{10}(0.05/(220 \times 164))$*. Traits are ordered by decreasing significance of the meta-analyzed p-values for CNS enrichment (Methods). All traits in this figure are classified as non-brain-related.*

bioRxiv preprint doi: https://doi.org/10.64898/2026.03.22.713538; this version posted March 25, 2026. The copyright holder for this preprint (which was not certified by peer review) is the author/funder, who has granted bioRxiv a license to display the preprint in perpetuity. It is made available under aCC-BY 4.0 International license.

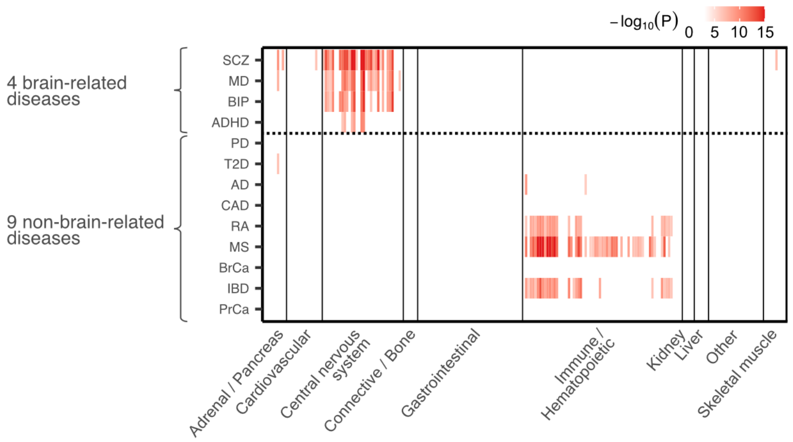

Figure S6: **Functional enrichment analyses with S-LDSC on 13 human complex diseases.** *Each row consists of 220 cell types from 10 categories, colored by* $-log_{10}(p)$ *of the coefficient* τ *in the S-LDSC model [26,27] if* $-log_{10}(p)$ *passes the Bonferroni correction threshold* $-log_{10}(0.05/(220 \times 164))$. *Traits are ordered by decreasing significance of the meta-analyzed p-values for CNS enrichment (Methods). The top 4 traits pass the significance threshold of* $-log_{10}(0.05/(10 \times 164))$ *and are therefore classified as brain-related. Disease full names can be found in Supplementary Table 2.*

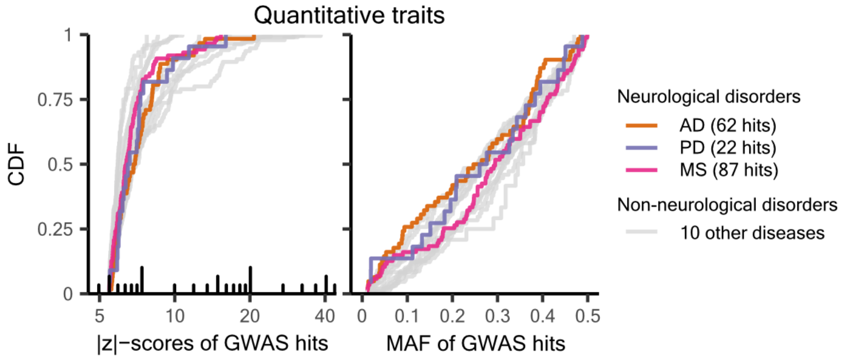

Figure S7: **Contrasting the GWAS hits for neurological diseases with those for 10 other human complex diseases.** *The full list of diseases can be found in Supplementary Table 2. AD: Alzheimer's disease; PD: Parkinson's disease; MS: multiple sclerosis.*

bioRxiv preprint doi: https://doi.org/10.64898/2026.03.22.713538; this version posted March 25, 2026. The copyright holder for this preprint (which was not certified by peer review) is the author/funder, who has granted bioRxiv a license to display the preprint in perpetuity. It is made available under aCC-BY 4.0 International license.

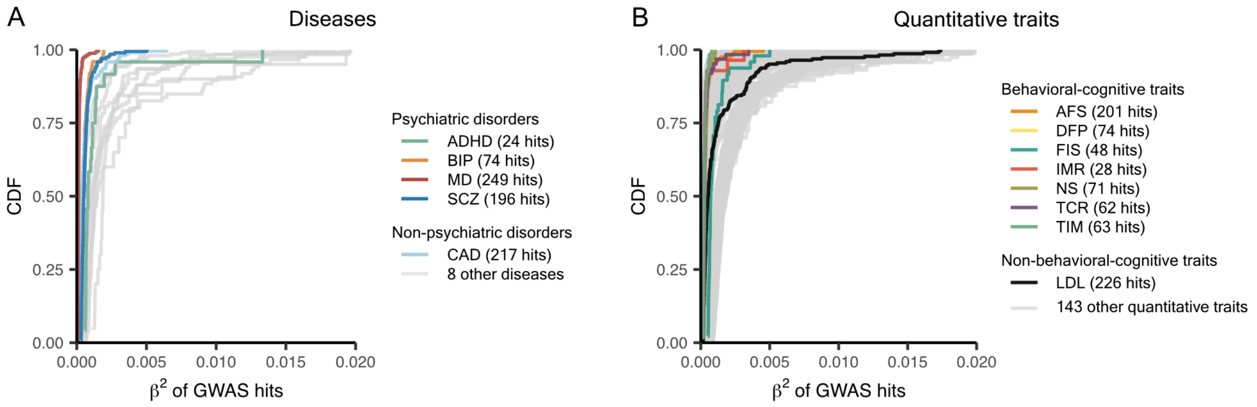

Figure S8: **Squared effect sizes of GWAS hits for brain-related versus non–brain-related traits.** ***A)*** *Psychiatric disorders compared with nine other complex diseases. Effect sizes reported on the log odds ratio scale (ζ) are transformed to the liability scale (β) with Equation 6. Details of the derivation are provided in the Supplementary Notes. When applying Equation 6, we assumed disease prevalence as reported in Supplementary Table 3.* ***B)*** *Behavioral-cognitive traits compared with 144 other quantitative traits.*

bioRxiv preprint doi: https://doi.org/10.64898/2026.03.22.713538; this version posted March 25, 2026. The copyright holder for this preprint (which was not certified by peer review) is the author/funder, who has granted bioRxiv a license to display the preprint in perpetuity. It is made available under aCC-BY 4.0 International license.

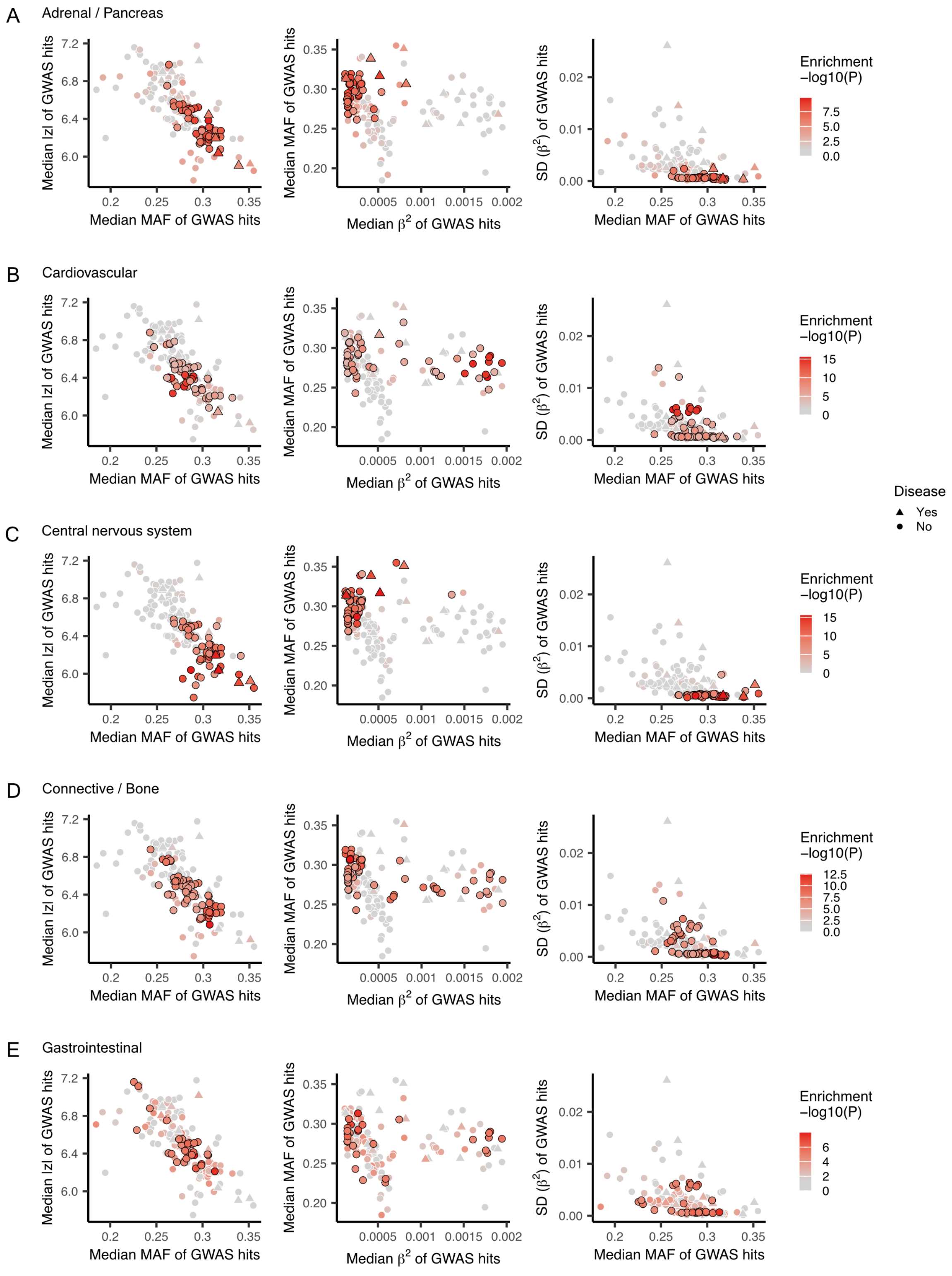

Figure S9: **Parameterizing the genetic architectures of complex traits (first half).** *Pairwise combinations of four GWAS hit metrics — median MAF, median $|z|$-score, median β^2, and standard deviation of β^2 — were used to parameterize trait architectures. All 164 traits included in this study are colored by meta-analyzed S-LDSC p-values for functional enrichment in the* ***A)*** *Adrenal / Pancreas* ***B)*** *Cardiovascular* ***C)*** *Central nervous system* ***D)*** *Connective / Bone* ***E)*** *Gastrointestinal category. Traits with p-values exceeding the significance threshold of* $0.05/(10 \times 164)$ *are outlined with black borders.*

bioRxiv preprint doi: https://doi.org/10.64898/2026.03.22.713538; this version posted March 25, 2026. The copyright holder for this preprint (which was not certified by peer review) is the author/funder, who has granted bioRxiv a license to display the preprint in perpetuity. It is made available under aCC-BY 4.0 International license.

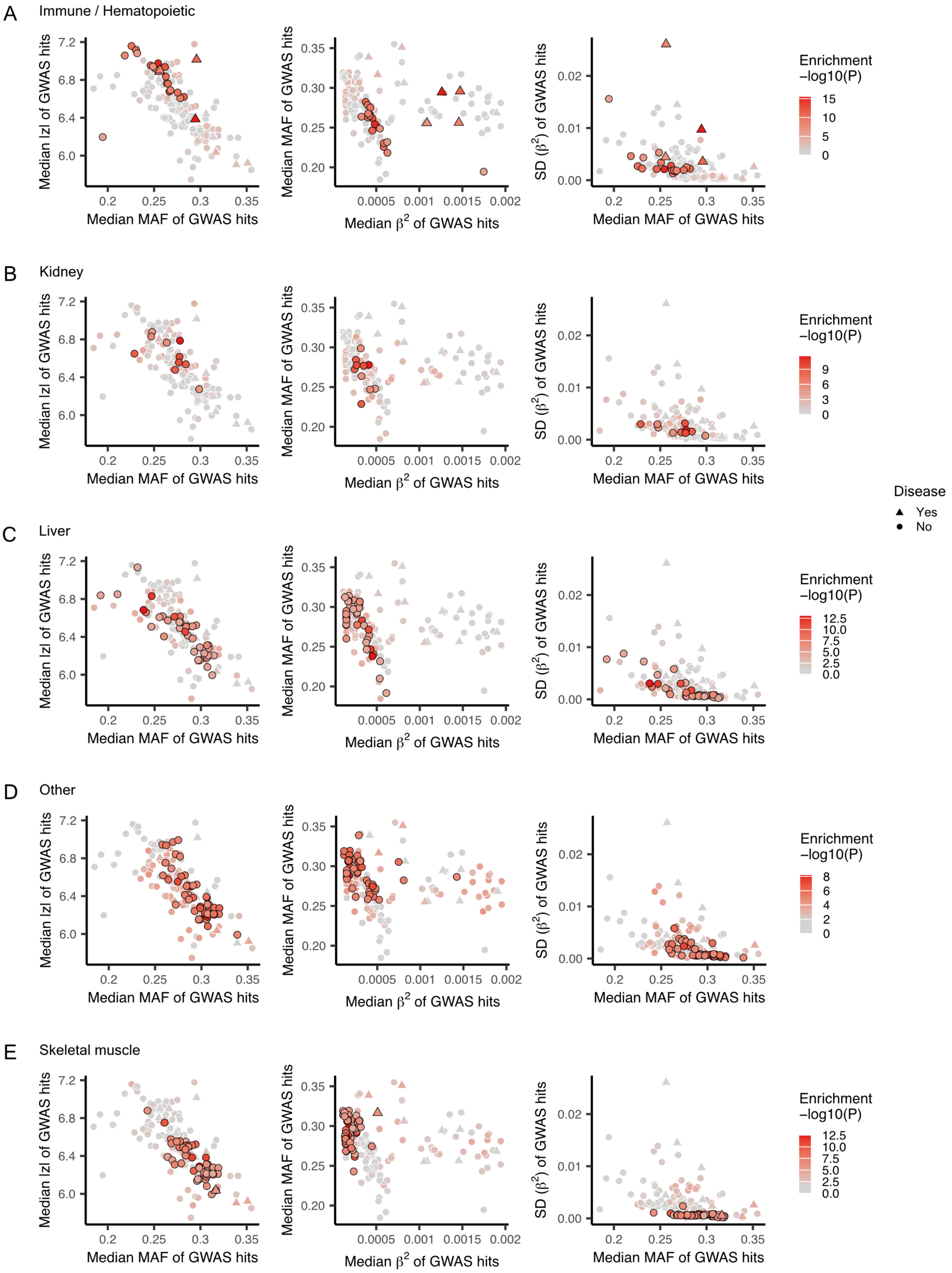

Figure S10: **Parameterizing the genetic architectures of complex traits (second half).** *Pairwise combinations of four GWAS hit metrics — median MAF, median* $|z|$*-score, median* β^2*, and standard deviation of* β^2 *— were used to parameterize trait architectures. All 164 traits included in this study are colored by meta-analyzed S-LDSC p-values for functional enrichment in the* ***A)*** *Immune / Hematopoietic* ***B)*** *Kidney* ***C)*** *Liver* ***D)*** *Other* ***E)*** *Skeletal muscle category. Traits with p-values exceeding the significance threshold of* $0.05/(10 \times 164)$ *are outlined with black borders.*

bioRxiv preprint doi: https://doi.org/10.64898/2026.03.22.713538; this version posted March 25, 2026. The copyright holder for this preprint (which was not certified by peer review) is the author/funder, who has granted bioRxiv a license to display the preprint in perpetuity. It is made available under aCC-BY 4.0 International license.

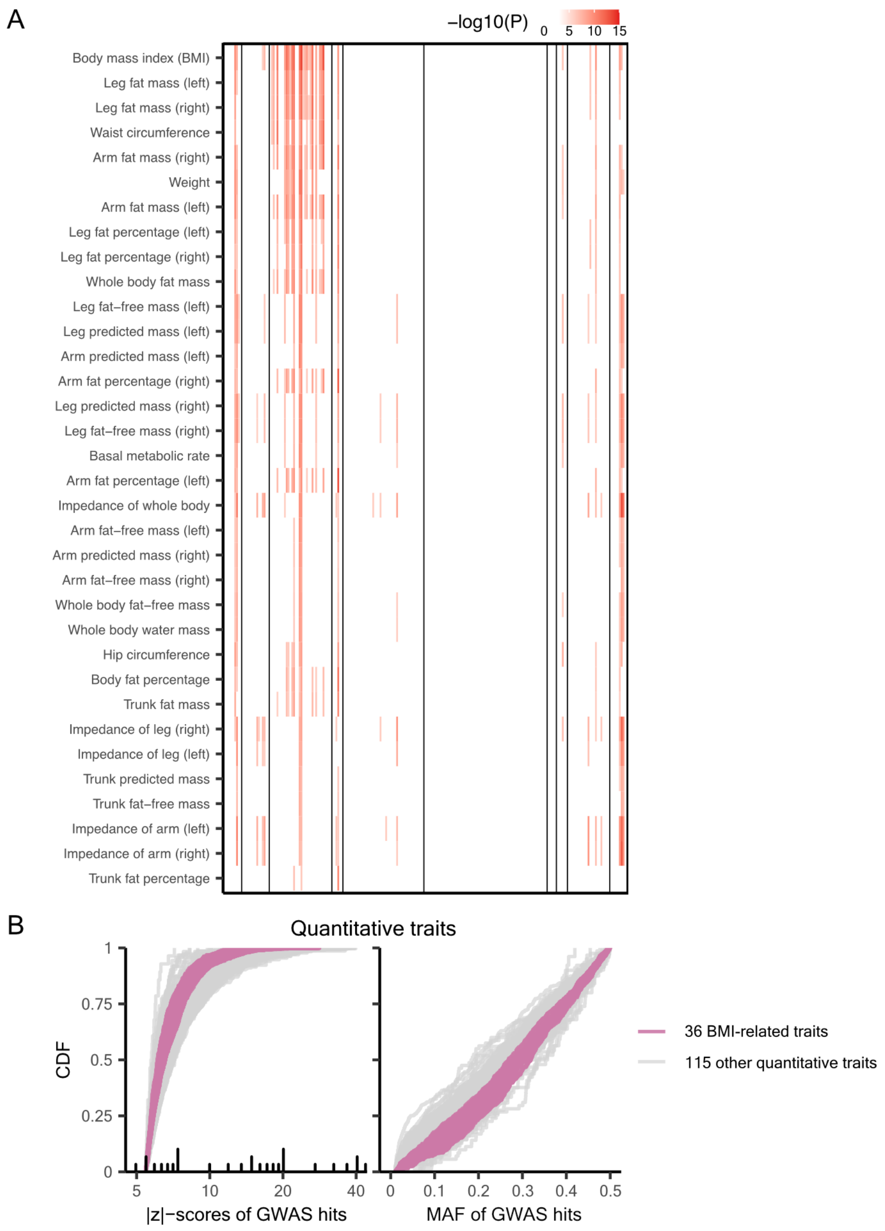

Figure S11: **Contrasting body composition-related traits with other quantitative traits.**
A) *S-LDSC profiles of the 36 body composition-related traits extracted from Figure S4.* ***B)*** *CDF curves for GWAS hits. The UK Biobank data fields for the 36 body composition–related traits are 48, 49, 21001, 21002, 23098–23102, and 23104–23130. Gray lines correspond to the rest of the 115 quantitative traits listed in Supplementary Table 1.*

bioRxiv preprint doi: https://doi.org/10.64898/2026.03.22.713538; this version posted March 25, 2026. The copyright holder for this preprint (which was not certified by peer review) is the author/funder, who has granted bioRxiv a license to display the preprint in perpetuity. It is made available under aCC-BY 4.0 International license.

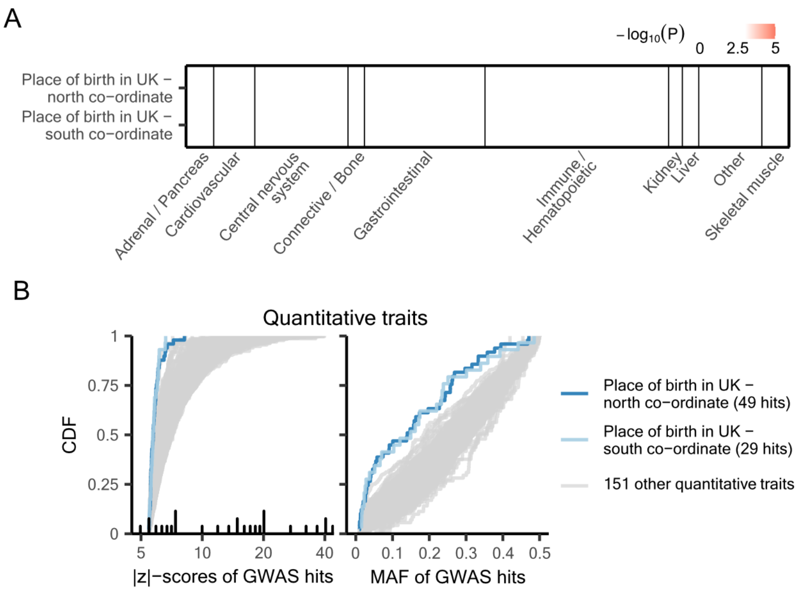

Figure S12: **Contrasting two birth coordinate traits with 151 selected quantitative traits.** ***A)*** *S-LDSC enrichment results.* ***B)*** *CDF curves for GWAS hits. UK Biobank data fields are 129 for Place of birth in UK - north co-ordinate and 130 for Place of birth in UK - south co-ordinate. Same as the other quantitative traits, GWAS summary statistics for these two birth coordinate traits were downloaded from the Neale Lab [35], with 20 genotyping PCs included as covariates. The full list of quantitative traits can be found in Supplementary Table 1.*

bioRxiv preprint doi: https://doi.org/10.64898/2026.03.22.713538; this version posted March 25, 2026. The copyright holder for this preprint (which was not certified by peer review) is the author/funder, who has granted bioRxiv a license to display the preprint in perpetuity. It is made available under aCC-BY 4.0 International license.

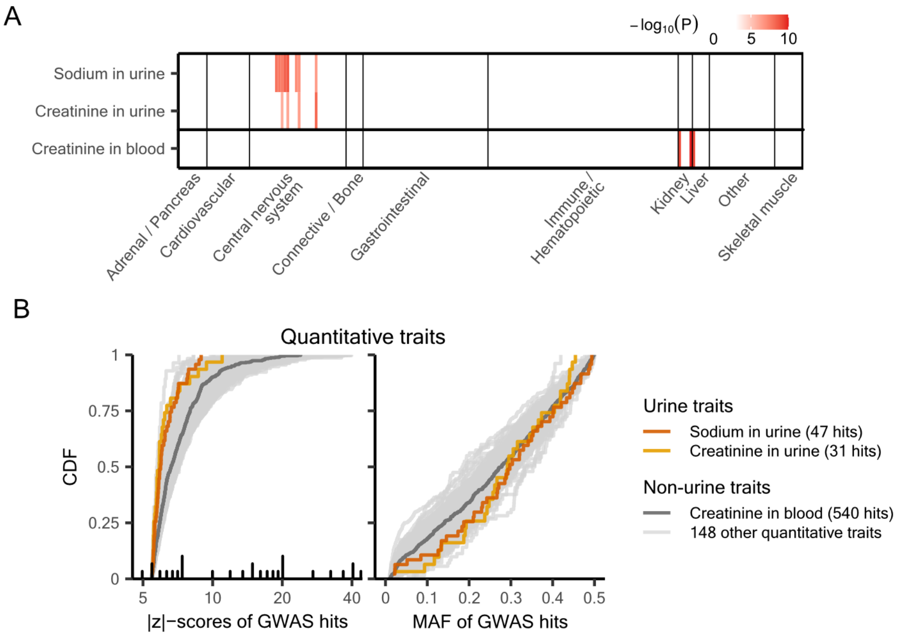

Figure S13: **Contrasting urinary traits with serum creatinine (and 148 other quantitative traits).** *A) S-LDSC enrichment results. B) CDF curves for GWAS hits. The full list of quantitative traits can be found in Supplementary Table 1. UK Biobank data fields are 30530 for sodium in urine, 30510 for creatinine in urine, and 30700 for creatinine in blood.*

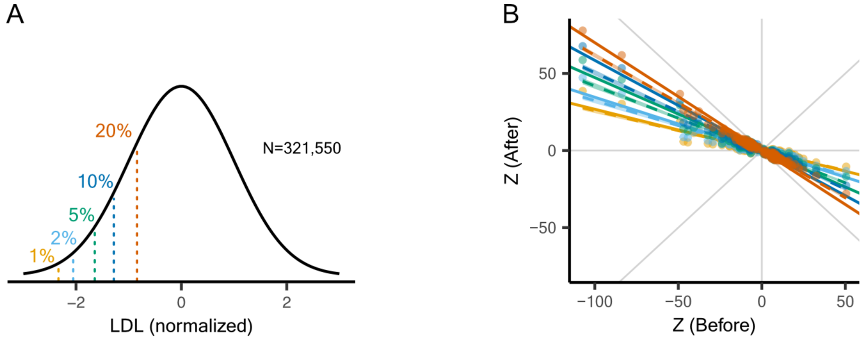

Figure S14: **LDL GWAS power reduction after binarization.**
***A)** Binarizing LDL levels with varying prevalence in the lower tail. **B)** Deflation of GWAS hit Z scores following lower-tail binarization of LDL. Different colors correspond to different prevalence used in panel A. Solid lines represent predicted slopes, and dashed lines indicate fitted slopes. Gray lines in the background are $y = 0$, $x = 0$ and $y = \pm x$. LDL was measured in unrelated White British individuals from the UK Biobank (data field 30780). The original phenotypic distribution was standardized using rank-based inverse normal transformation.*

bioRxiv preprint doi: https://doi.org/10.64898/2026.03.22.713538; this version posted March 25, 2026. The copyright holder for this preprint (which was not certified by peer review) is the author/funder, who has granted bioRxiv a license to display the preprint in perpetuity. It is made available under aCC-BY 4.0 International license.

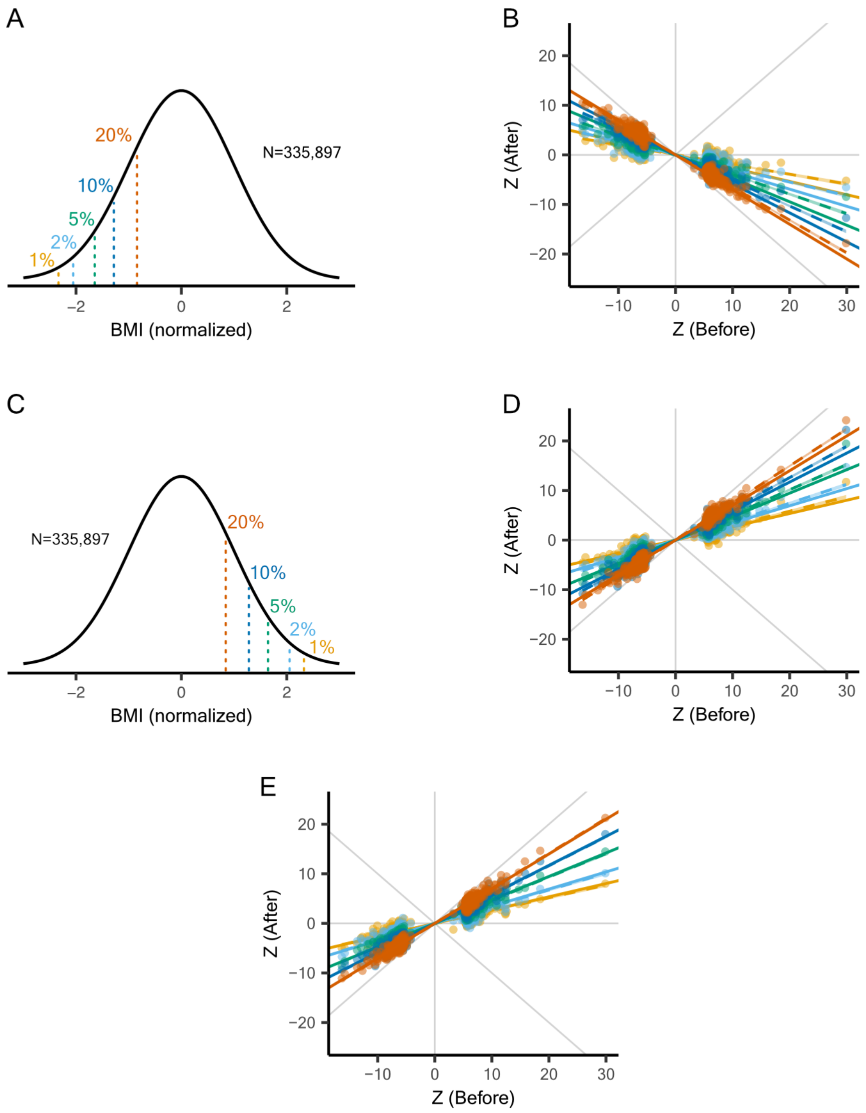

Figure S15: **BMI GWAS power reduction after binarization or downsampling.**
Binarizing BMI with varying prevalence in the ***A)*** *lower tail or the* ***C)*** *upper tail. Deflation of GWAS hit Z scores after* ***B)*** *binarization in the lower tail,* ***D)*** *binarization in the upper tail, or* ***E)*** *downsampling BMI to matched sample sizes. In panels B, D and E, different colors correspond to different prevalence used in panels A and C. Solid lines represent predicted slopes, and dashed lines indicate fitted slopes. Gray lines in the background are* $y = 0$, $x = 0$ *and* $y = \pm x$. *BMI was measured in unrelated White British individuals from the UK Biobank (data field 21001). The original phenotypic distribution was standardized using rank-based inverse normal transformation.*

bioRxiv preprint doi: https://doi.org/10.64898/2026.03.22.713538; this version posted March 25, 2026. The copyright holder for this preprint (which was not certified by peer review) is the author/funder, who has granted bioRxiv a license to display the preprint in perpetuity. It is made available under aCC-BY 4.0 International license.

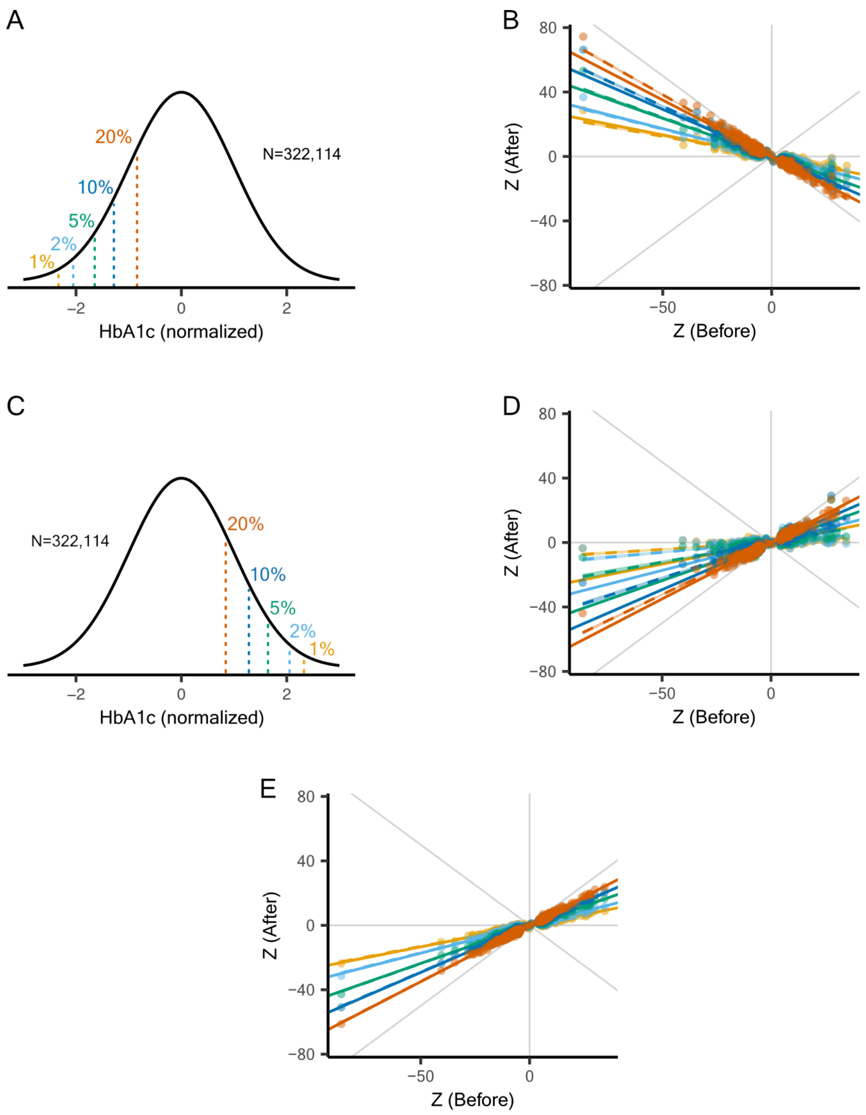

Figure S16: **HbA1c GWAS power reduction after binarization or downsampling.** *Binarizing hemoglobin A1C (HbA1c) with varying prevalence in the* ***A)*** *lower tail or the* ***C)*** *upper tail. Deflation of GWAS hit Z scores after* ***B)*** *binarization in the lower tail,* ***D)*** *binarization in the upper tail, or* ***E)*** *downsampling HbA1c to matched sample sizes. In panels B, D and E, different colors correspond to different prevalence used in panels A and C. Solid lines represent predicted slopes, and dashed lines indicate fitted slopes. Gray lines in the background are* $y = 0$, $x = 0$ *and* $y = \pm x$. *HbA1c was measured in unrelated White British individuals from the UK Biobank (data field 30750). The original phenotypic distribution was standardized using rank-based inverse normal transformation.*

bioRxiv preprint doi: https://doi.org/10.64898/2026.03.22.713538; this version posted March 25, 2026. The copyright holder for this preprint (which was not certified by peer review) is the author/funder, who has granted bioRxiv a license to display the preprint in perpetuity. It is made available under aCC-BY 4.0 International license.

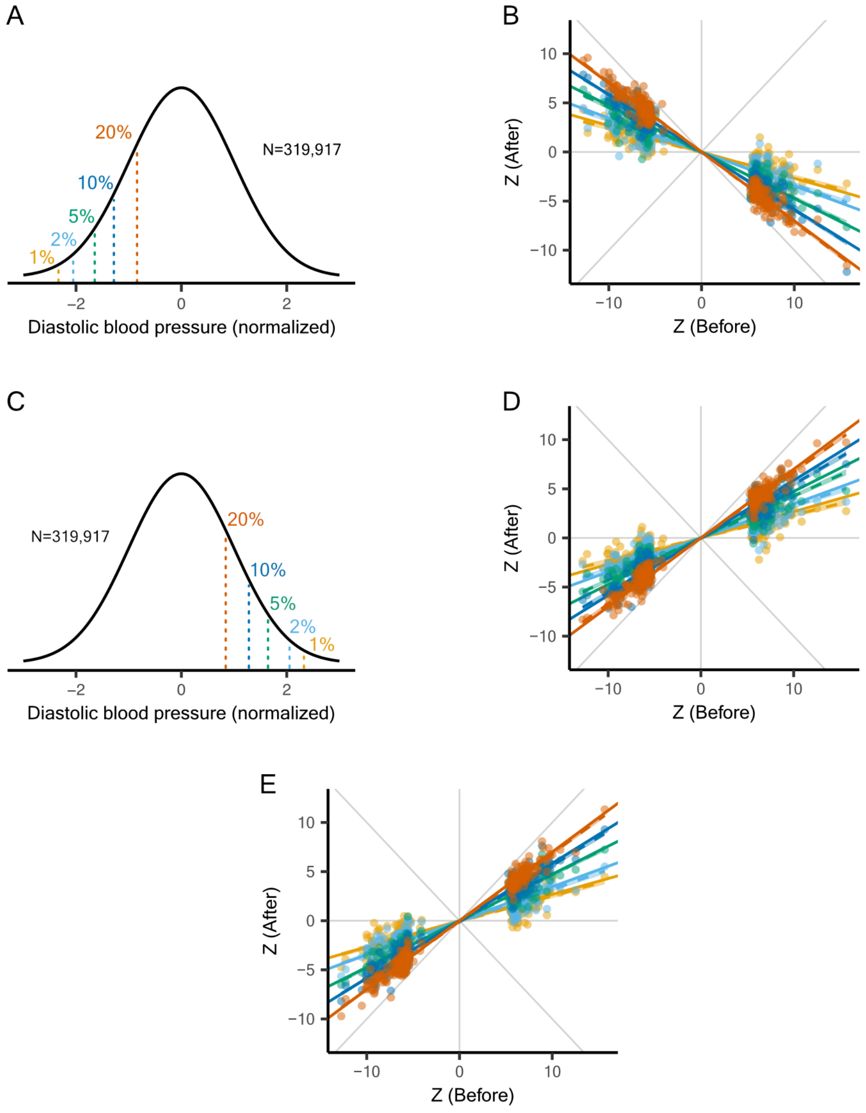

Figure S17: **Diastolic blood pressure GWAS power reduction after binarization or downsampling.** *Binarizing diastolic blood pressure with varying prevalence in the* ***A)*** *lower tail or the* ***C)*** *upper tail. Deflation of GWAS hit Z scores after* ***B)*** *binarization in the lower tail,* ***D)*** *binarization in the upper tail, or* ***E)*** *downsampling diastolic blood pressure to matched sample sizes. In panels B, D and E, different colors correspond to different prevalence used in panels A and C. Solid lines represent predicted slopes, and dashed lines indicate fitted slopes. Gray lines in the background are $y = 0$, $x = 0$ and $y = \pm x$. Diastolic blood pressure was measured in unrelated White British individuals from the UK Biobank (data field 4079). The original phenotypic distribution was standardized using rank-based inverse normal transformation.*

bioRxiv preprint doi: https://doi.org/10.64898/2026.03.22.713538; this version posted March 25, 2026. The copyright holder for this preprint (which was not certified by peer review) is the author/funder, who has granted bioRxiv a license to display the preprint in perpetuity. It is made available under aCC-BY 4.0 International license.

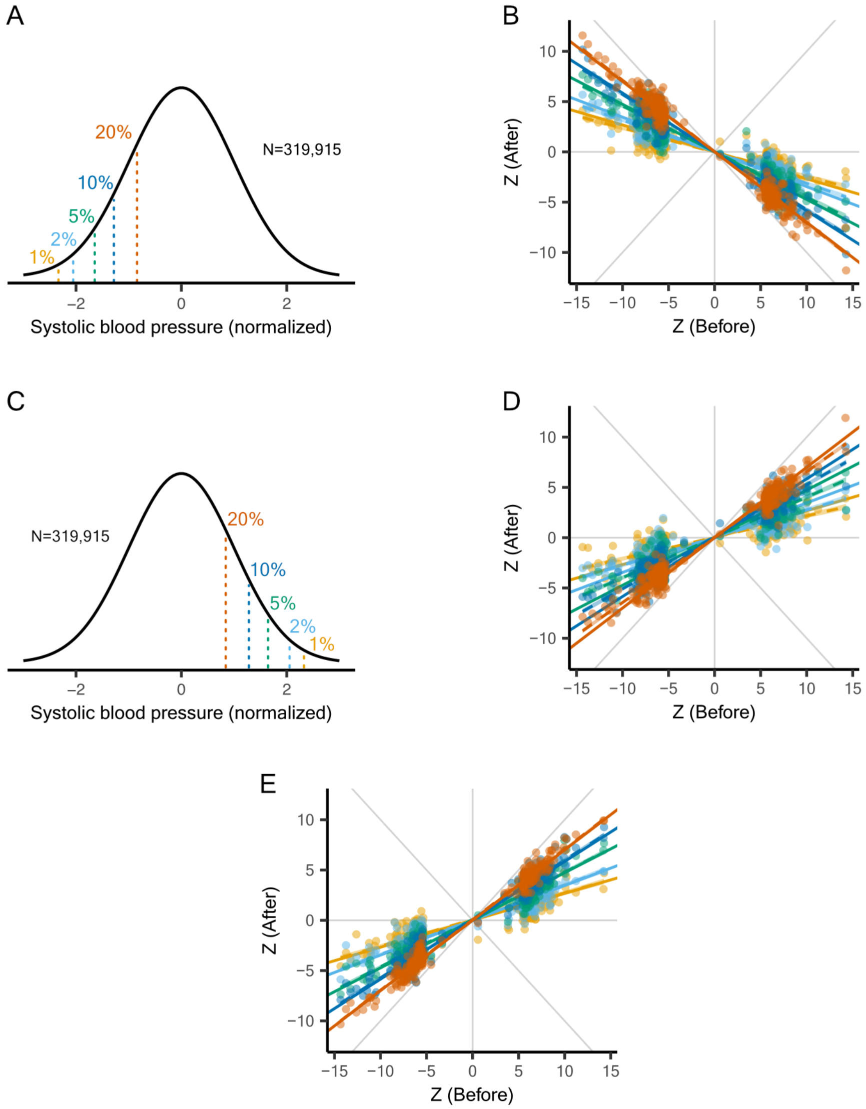

Figure S18: **Systolic blood pressure GWAS power reduction after binarization or downsampling.** *Binarizing systolic blood pressure with varying prevalence in the* ***A)*** *lower tail or the* ***C)*** *upper tail. Deflation of GWAS hit Z scores after* ***B)*** *binarization in the lower tail,* ***D)*** *binarization in the upper tail, or* ***E)*** *downsampling systolic blood pressure to matched sample sizes. In panels B, D and E, different colors correspond to different prevalence used in panels A and C. Solid lines represent predicted slopes, and dashed lines indicate fitted slopes. Gray lines in the background are* $y = 0$, $x = 0$ *and* $y = \pm x$. *Systolic blood pressure was measured in unrelated White British individuals from the UK Biobank (data field 4080). The original phenotypic distribution was standardized using rank-based inverse normal transformation.*

bioRxiv preprint doi: https://doi.org/10.64898/2026.03.22.713538; this version posted March 25, 2026. The copyright holder for this preprint (which was not certified by peer review) is the author/funder, who has granted bioRxiv a license to display the preprint in perpetuity. It is made available under aCC-BY 4.0 International license.

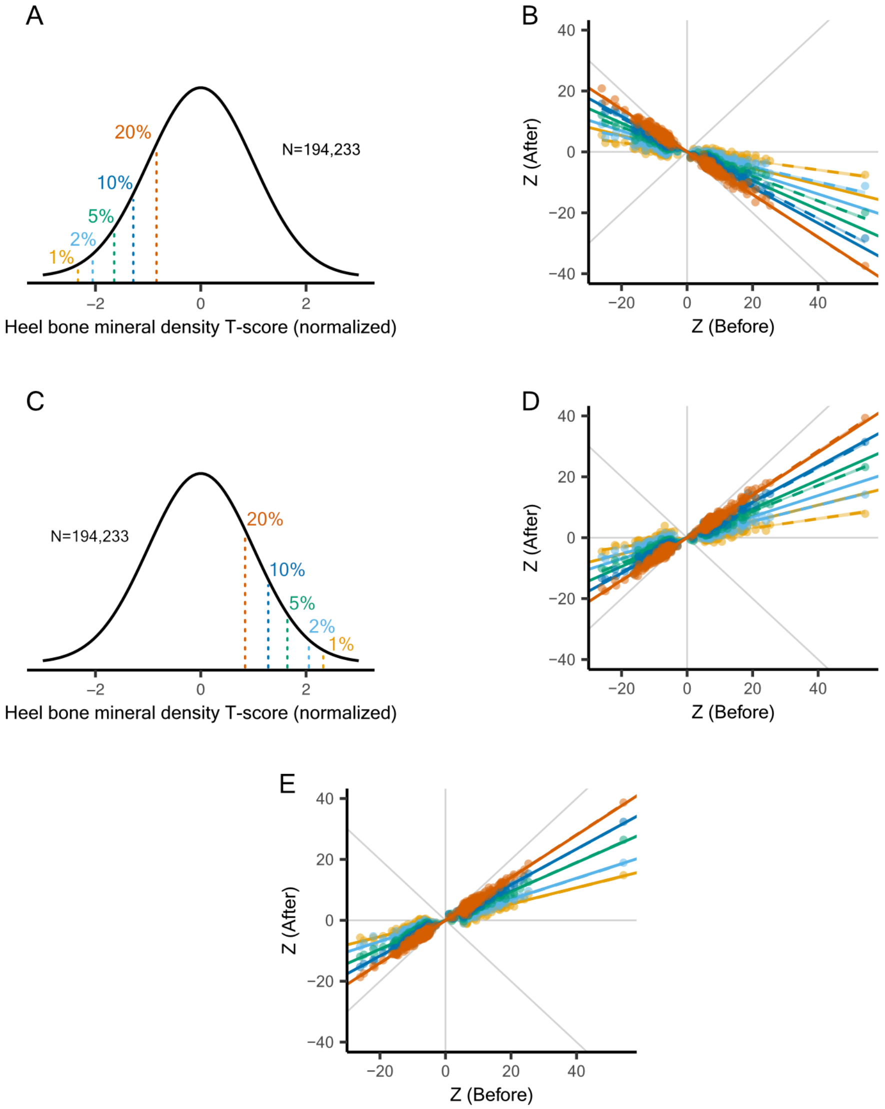

Figure S19: **Heel bone mineral density T-score GWAS power reduction after binarization or downsampling.** *Binarizing heel bone mineral density T-score with varying prevalence in the **A)** lower tail or the **C)** upper tail. Deflation of GWAS hit Z scores after **B)** binarization in the lower tail, **D)** binarization in the upper tail, or **E)** downsampling heel bone mineral density T-score to matched sample sizes. In panels B, D and E, different colors correspond to different prevalence used in panels A and C. Solid lines represent predicted slopes, and dashed lines indicate fitted slopes. Gray lines in the background are $y = 0$, $x = 0$ and $y = \pm x$. Heel bone mineral density T-score was measured in unrelated White British individuals from the UK Biobank (data field 78). The original phenotypic distribution was standardized using rank-based inverse normal transformation.*

bioRxiv preprint doi: https://doi.org/10.64898/2026.03.22.713538; this version posted March 25, 2026. The copyright holder for this preprint (which was not certified by peer review) is the author/funder, who has granted bioRxiv a license to display the preprint in perpetuity. It is made available under aCC-BY 4.0 International license.

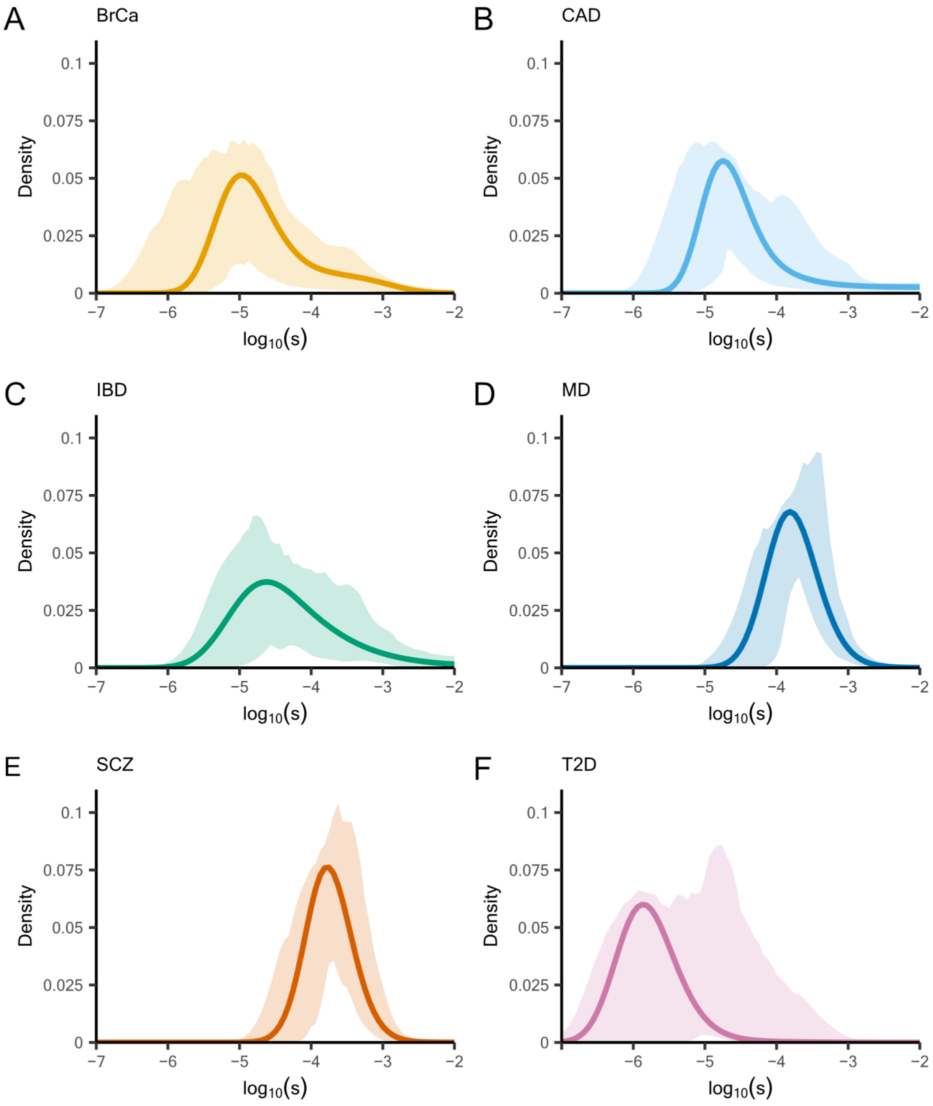

Figure S20: **Trait-specific distributions of selection coefficients inferred from** ***A)*** *breast cancer* ***B)*** *coronary artery disease* ***C)*** *inflammatory bowel disease* ***D)*** *major depression* ***E)*** *schizophrenia* ***F)*** *type 2 diabetes GWAS hits, with 90% confidence envelopes shown.*

bioRxiv preprint doi: https://doi.org/10.64898/2026.03.22.713538; this version posted March 25, 2026. The copyright holder for this preprint (which was not certified by peer review) is the author/funder, who has granted bioRxiv a license to display the preprint in perpetuity. It is made available under aCC-BY 4.0 International license.

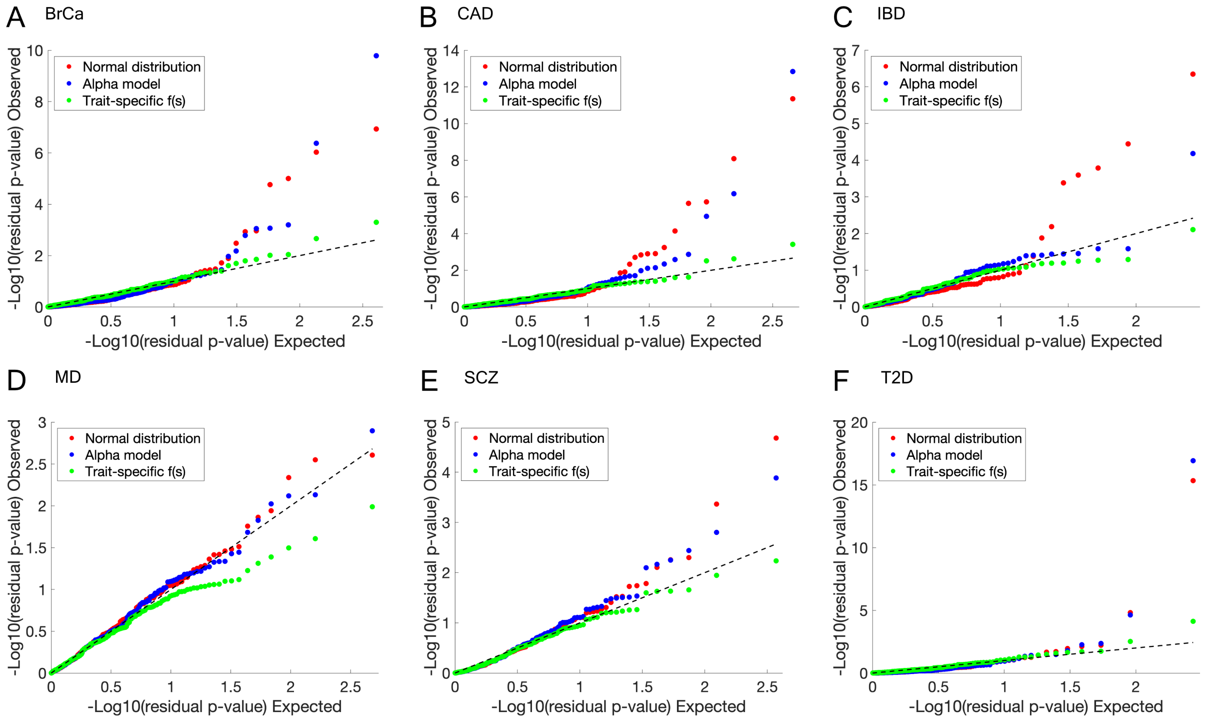

Figure S21: **QQ plots of residual p-values for** ***A)*** *breast cancer* ***B)*** *coronary artery disease* ***C)*** *inflammatory bowel disease* ***D)*** *major depression* ***E)*** *schizophrenia* ***F)*** *type 2 diabetes,* **evaluated under three models: the normal distribution, the alpha model, and the adapted Simons model, which infers trait-specific $f(s)$.**

bioRxiv preprint doi: https://doi.org/10.64898/2026.03.22.713538; this version posted March 25, 2026. The copyright holder for this preprint (which was not certified by peer review) is the author/funder, who has granted bioRxiv a license to display the preprint in perpetuity. It is made available under aCC-BY 4.0 International license.

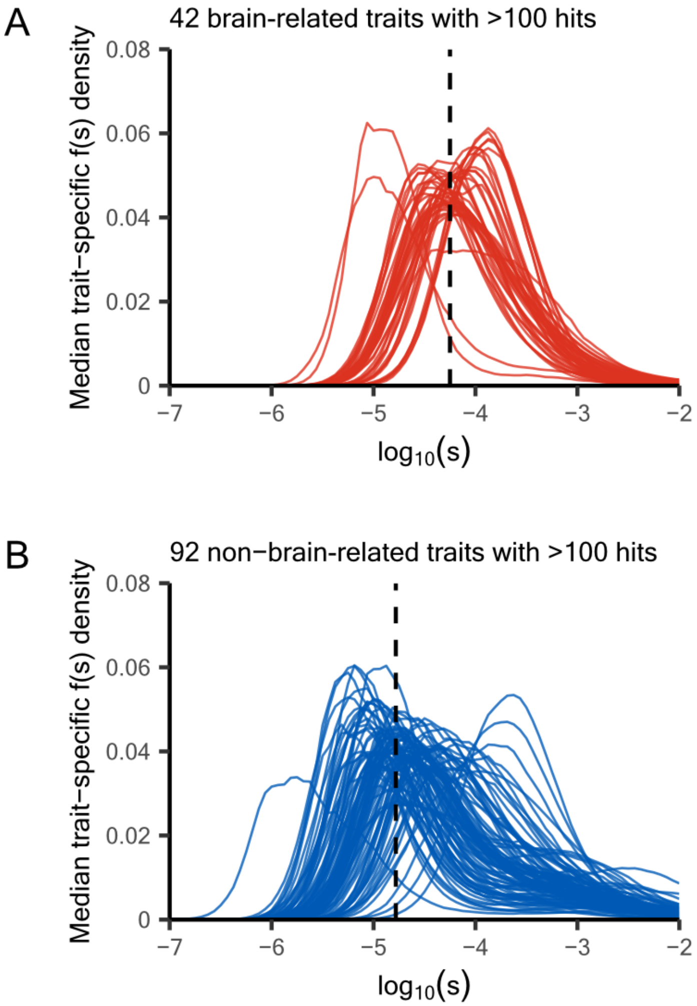

Figure S22: **Median trait-specific distributions of selection coefficients inferred from 134 traits with at least 100 GWAS hits, with traits grouped into** ***A)*** *brain-related* **versus** ***B)*** *non-brain-related* **traits.** *Vertical line in each plot correspond to the median mode.*

bioRxiv preprint doi: https://doi.org/10.64898/2026.03.22.713538; this version posted March 25, 2026. The copyright holder for this preprint (which was not certified by peer review) is the author/funder, who has granted bioRxiv a license to display the preprint in perpetuity. It is made available under aCC-BY 4.0 International license.

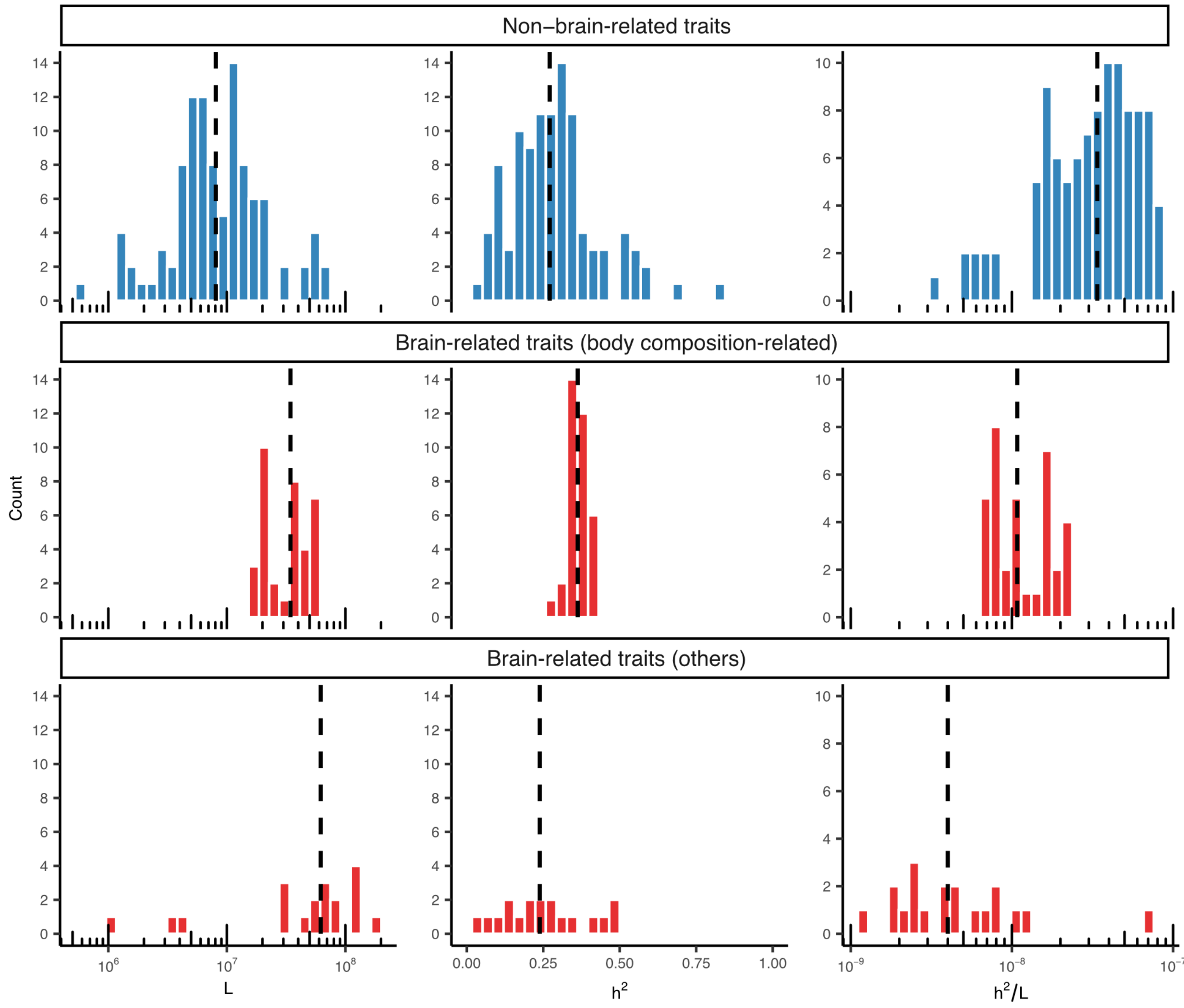

Figure S23: **Re-plotting the estimates in Figure 4D as histograms to highlight distribution shifts.** *Dashed vertical lines indicate the median values in each subplot. Brain-related traits are further divided into body composition-related traits and other brain-related traits. Body composition-related traits have L and h^2/L estimates that fall between those of non–brain-related traits and traits primarily influenced by the CNS alone.*

bioRxiv preprint doi: https://doi.org/10.64898/2026.03.22.713538; this version posted March 25, 2026. The copyright holder for this preprint (which was not certified by peer review) is the author/funder, who has granted bioRxiv a license to display the preprint in perpetuity. It is made available under aCC-BY 4.0 International license.

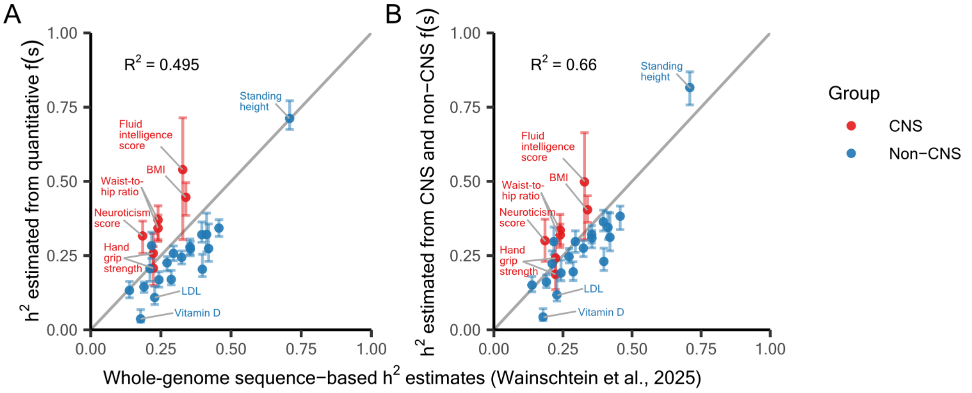

Figure S24: **Comparing heritability estimates obtained in this study with recently reported whole-genome sequence-based heritability estimates (h^2_{WGS}).**
A) *Comparing h^2_{WGS} with h^2 obtained jointly with quantitative $f(s)$, as shown in Figure 4B.* ***B)*** *Comparing h^2_{WGS} with h^2 obtained jointly with brain-related or non-brain-related $f(s)$, as shown in Figure 4C. Wainschtein et al. [57] estimated h^2_{WGS} in 347,630 unrelated individuals from the UK Biobank with GREML-LDMS .*

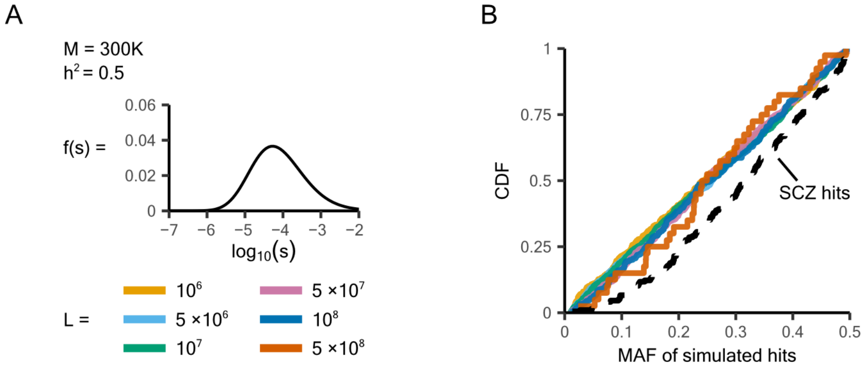

Figure S25: **Variation in MAF distributions of GWAS hits cannot be recovered by changing L.**
A) *Same simulation setup as in Figure 5A.* ***B)*** *Varying L does not change the MAF distribution of GWAS hits. The black dashed line shows real schizophrenia GWAS hits for reference.*

bioRxiv preprint doi: https://doi.org/10.64898/2026.03.22.713538; this version posted March 25, 2026. The copyright holder for this preprint (which was not certified by peer review) is the author/funder, who has granted bioRxiv a license to display the preprint in perpetuity. It is made available under aCC-BY 4.0 International license.

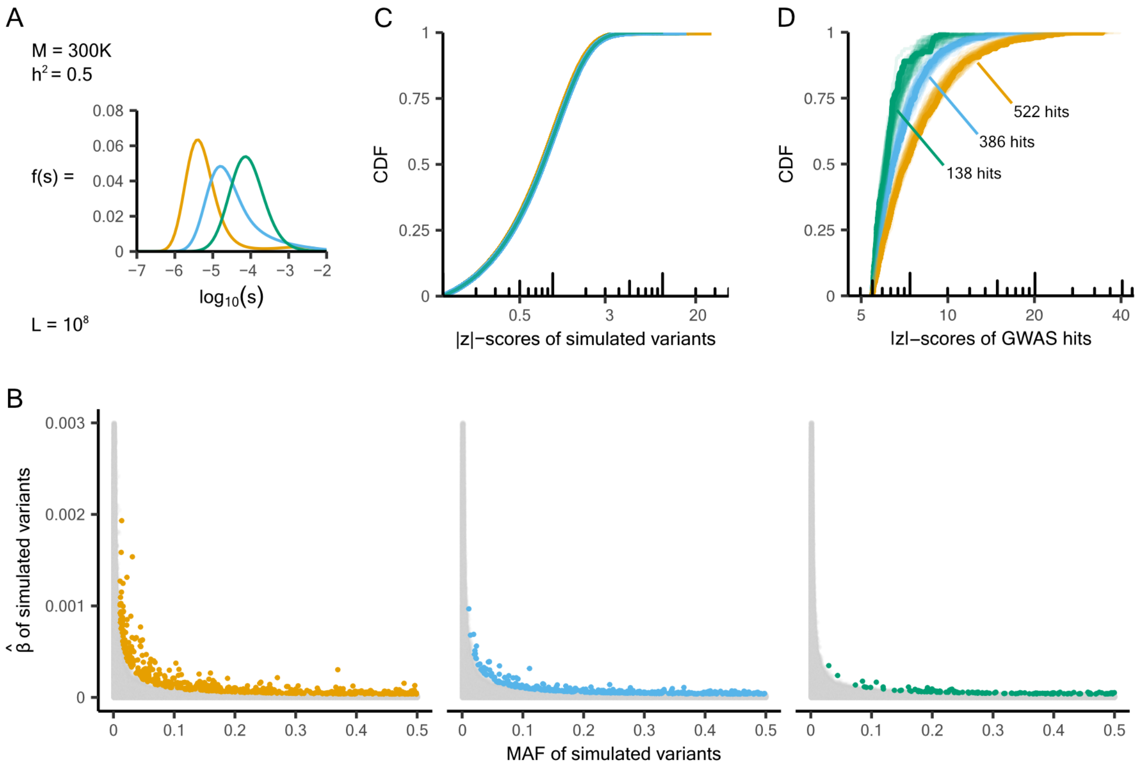

Figure S26: **Impact of GWAS ascertainment on simulations under different $f(s)$ distributions.** ***A)*** *Same simulation setup as in Figure 5D.* ***B)*** *The GWAS ascertainment process. Variants with MAF $> 1\%$ and $|Z| > 5.45$ are discovered as hits and colored according to the $f(s)$ distribution from which they were simulated. Unselected variants are shown in grey.* ***C)*** *Without GWAS ascertainment, the CDFs of $|Z|$ scores for variants simulated under different $f(s)$ distributions differ in the tails.* ***D)*** *After GWAS ascertainment, stronger $f(s)$ produces steeper GWAS hit $|Z|$ score CDFs and fewer hits.*

bioRxiv preprint doi: https://doi.org/10.64898/2026.03.22.713538; this version posted March 25, 2026. The copyright holder for this preprint (which was not certified by peer review) is the author/funder, who has granted bioRxiv a license to display the preprint in perpetuity. It is made available under aCC-BY 4.0 International license.

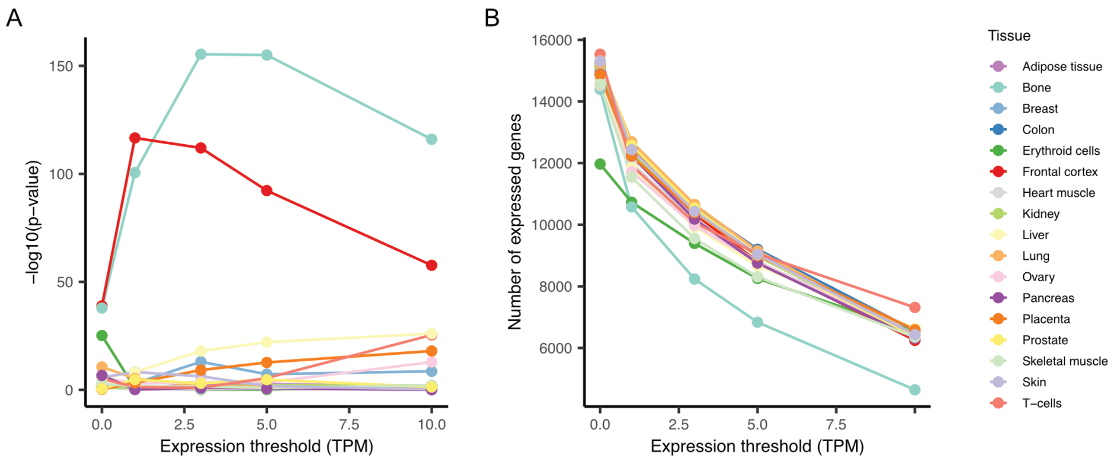

Figure S27: **Brain expression status strongly predicts a gene's constraint estimate, s_{het}.** ***A)*** *P-values from multiple regression models, where $\log_{10}(s_{het})$ is regressed on expression status across 17 tissues. A gene is classified as "expressed" in a given tissue if its transcripts per million (TPM) values exceeds a threshold of 0, 1, 3, 5, or 10.* ***B)*** *While "frontal cortex" and "bone" appear to be the strongest predictors in panel A, genes are generally lowly expressed in the bone, and increasing the TPM cutoff rapidly excludes a large number of genes. In contrast, expression levels in the frontal cortex are more comparable to those in other tissues, indicating that its signal in panel A is not driven by a more rapid loss of genes at higher TPM thresholds. Gene expression values across tissues were curated in [39], with data extracted from the Human Protein Atlas [99].*

bioRxiv preprint doi: https://doi.org/10.64898/2026.03.22.713538; this version posted March 25, 2026. The copyright holder for this preprint (which was not certified by peer review) is the author/funder, who has granted bioRxiv a license to display the preprint in perpetuity. It is made available under aCC-BY 4.0 International license.

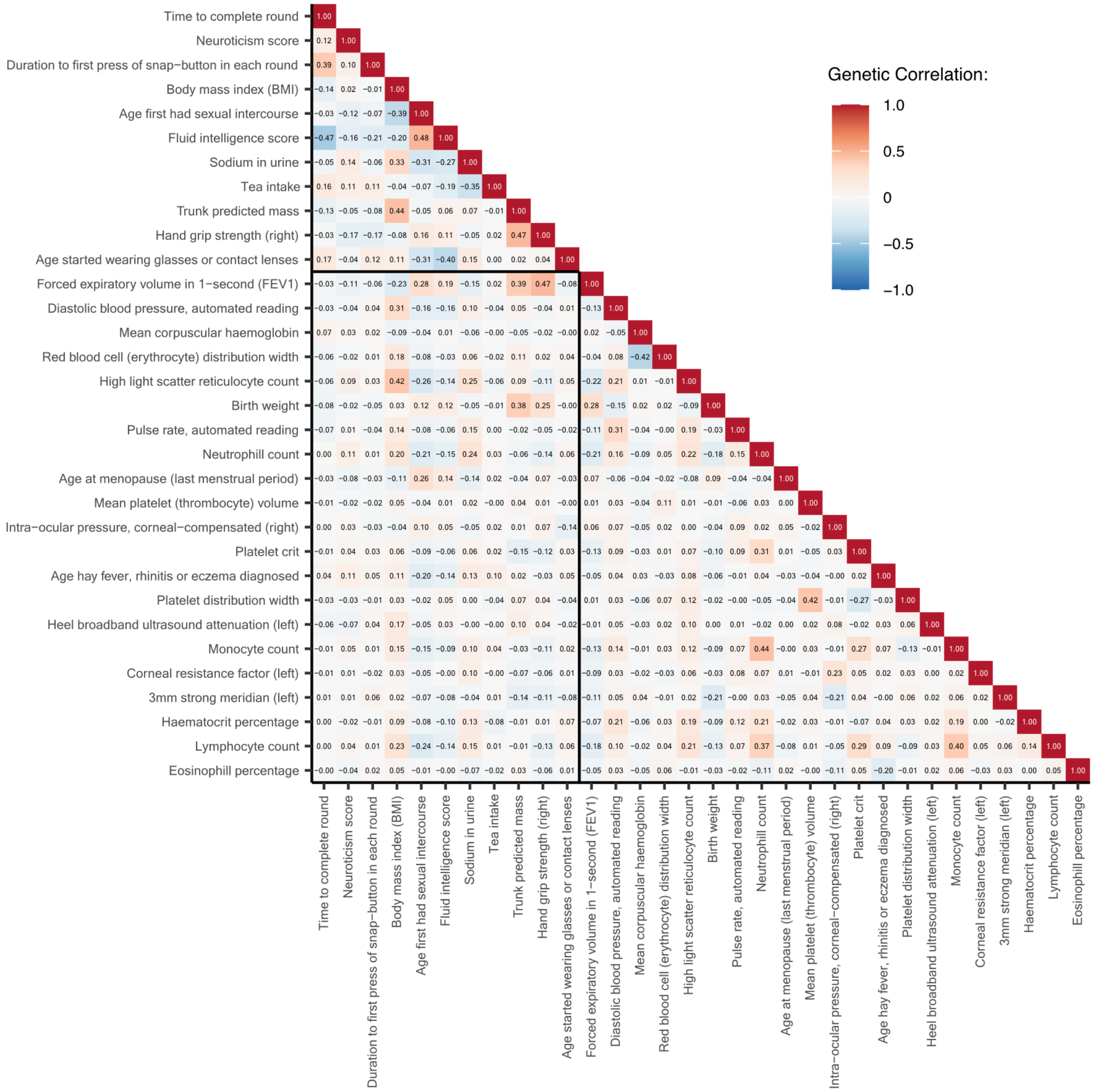

Figure S28: **Heatmap of pairwise genetic correlations across 32 independent traits.** *The top 11 traits are CNS traits.*

bioRxiv preprint doi: https://doi.org/10.64898/2026.03.22.713538; this version posted March 25, 2026. The copyright holder for this preprint (which was not certified by peer review) is the author/funder, who has granted bioRxiv a license to display the preprint in perpetuity. It is made available under aCC-BY 4.0 International license.

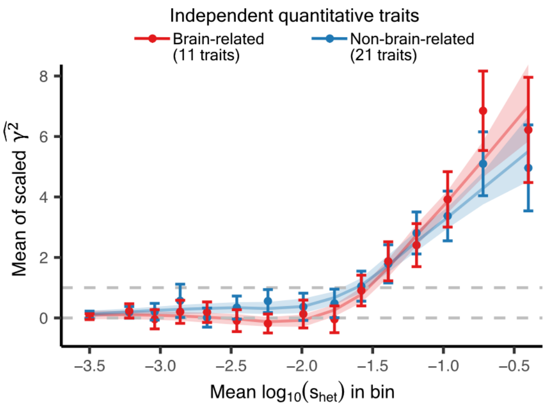

Figure S29: **Mean squared genic effects on independent brain-related versus non-brain-related traits.** *Genes are grouped into 15 quantiles of s_{het} estimates [58]. The estimated $\widehat{\gamma^2}$ values are scaled so that the mean across all gene-trait pairs in the tenth bin equals one. We applied a MAF < 1% filter. All estimates are shown with a 95% confidence interval. We bootstrapped genes at the trait level and showed the mean and 95% confidence intervals of the fitted LOESS curves.*

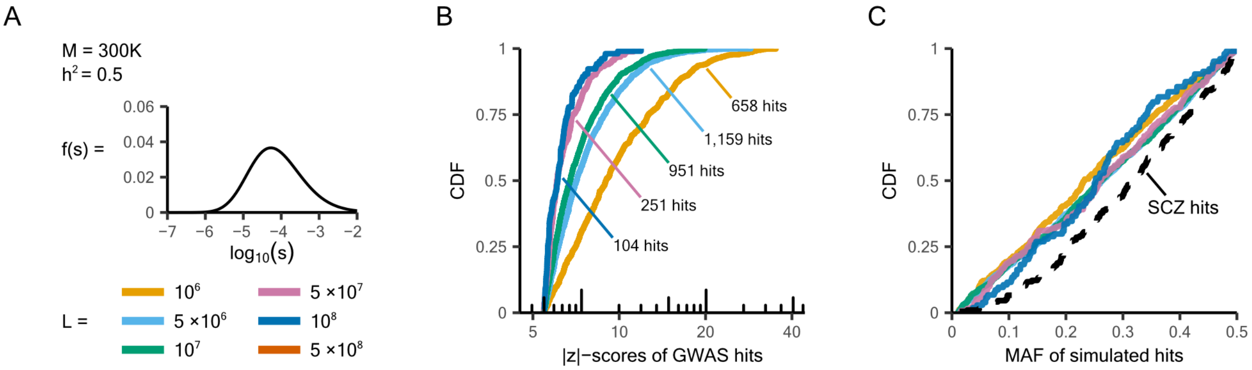

Figure S30: **Assuming no pleiotropy for variant effects fails to recapitulate the observed variation in MAF distributions of GWAS hits.**
A) *Simulating under a modified Simons model that assumes no pleiotropy (Methods). The rest of the simulation set up is the same as in Figure 5A and S25.* ***B)*** *Larger L values lead to narrower distributions of GWAS hit |z|-scores. Simulating with a mutational target size of 5×10^8 did not generate any GWAS hits.* ***C)*** *Varying L does not change the MAF distribution of GWAS hits. The black dashed line shows real schizophrenia GWAS hits for reference.*
